# Feasibility of wastewater-based detection of emergent pandemics through a global network of airports

**DOI:** 10.1371/journal.pgph.0003010

**Published:** 2024-03-13

**Authors:** Shihui Jin, Borame L. Dickens, Kai Yee Toh, David Chien Boon Lye, Vernon J. Lee, Alex R. Cook

**Affiliations:** 1 Saw Swee Hock School of Public Health, National University of Singapore and National University Health System, Singapore, Singapore; 2 AMILI Pte Ltd, Singapore, Singapore; 3 Department of Infectious Diseases, Tan Tock Seng Hospital, Singapore, Singapore; 4 Lee Kong Chian School of Medicine, Nanyang Technological University, Singapore, Singapore; 5 National Centre for Infectious Diseases, Singapore, Singapore; 6 Yong Loo Lin School of Medicine, National University of Singapore, Singapore, Singapore; 7 Ministry of Health, Singapore, Singapore; 8 Department of Statistics and Data Science, National University of Singapore, Singapore, Singapore; PLOS: Public Library of Science, UNITED STATES

## Abstract

Wastewater-based surveillance has been put into practice during the pandemic. Persistence of SARS-CoV-2 in faeces of infected individuals, and high volume of passengers travelling by air, make it possible to detect virus from aircraft wastewater, lending itself to the potential identification of a novel pathogen prior to clinical diagnosis. In this study, we estimated the likelihood of detecting the virus through aircraft wastewater from the probabilities of air travel, viral shedding, defecation, testing sensitivity, and sampling. We considered various hypothetical scenarios, with diverse sampling proportions of inbound flights, surveillance airports, and sources of outbreaks. Our calculations showed that the probability of detecting SARS-CoV-2 would increase exponentially against time in the early phase of the pandemic, and would be much higher if the 20 major airports in Asia, Europe, and North America cooperated to perform aircraft wastewater surveillance. We also found other contributors to early detection, including high sampling proportion of inbound flight at destination airports, small population size of the epicentre relative to the travel volume, and large volume of outbound travelers to major airports around the globe. We concluded that routine aircraft wastewater monitoring could be a feasible approach for early identification and tracking of an emerging pathogen with high faecal shedding rates, particularly when implemented through a global surveillance network of major airports.

## Introduction

As the coronavirus disease 2019 (COVID-19) pandemic evolves into an endemic state, attention is shifting to early detection of the next pandemic. For pathogens causing symptoms that are non-specific and sometimes mild, detection at the epicentre may be delayed, especially if there is lack of surveillance capability there, potentially limiting the ability to prevent the outbreak at source. Testing travelers for novel pathogens is one opportunity for global surveillance, but taking samples directly from sufficiently large numbers of passengers would be highly impractical considering current travel volumes and testing capacities at most airports.

Those infected with respiratory viral infections, including severe acute respiratory syndrome coronavirus 2 (SARS-CoV-2), have been found to shed viral RNA frequently in their faeces, apart from the respiratory tract [1, 2]. Furthermore, faecal viral shedding exists among different stages of infections—be they asymptomatic, pre-symptomatic, or symptomatic—and persists even after symptom recovery [3]. In fact, the viability of measuring disease prevalence through wastewater has been explored widely, and in many cases identification of the virus in wastewater samples contributed to surveillance of SARS-CoV-2 infection in a specific population [4, 5].

On the other hand, air travel facilitated the spread of COVID-19 from the epicentre to the rest of the world. A strong correlation has been found between passenger volume and importation of COVID-19 cases at different places, particularly countries or regions outside China that are less accessible by land travel [6, 7]. This suggests the promising use of aircraft wastewater surveillance to detect nascent transmission of a new virus and give an early warning of a future outbreak, although the exact prevalence may be under-estimated due to limited time spent on a plane and passengers’ infrequent toileting habits (especially for short-haul flights) [8].

Until now, there has been limited effort paid to quantitatively assessing the feasibility of detecting the presence of SARS-CoV-2, or some other emerging respiratory pathogen, in wastewater generated by passengers on board, despite the ample data available for analysis. To fill this gap, we constructed a probability model to retrospectively estimate the likelihoods of discerning the virus from wastewater of inbound flights at a diverse set of airports, utilising epidemiological information of SARS-CoV-2, defaecation habits, and air travel data. We considered various sampling strategies, as well as hypothetical scenarios with alternative epicentres, and calculated their respective detection probabilities against time from incursion of the outbreak. Our inference results indicate the potential practicability of testing aircraft wastewater for the identification of a virus in the early stage of an infectious disease outbreak.

## Methods

**Fig 1:**
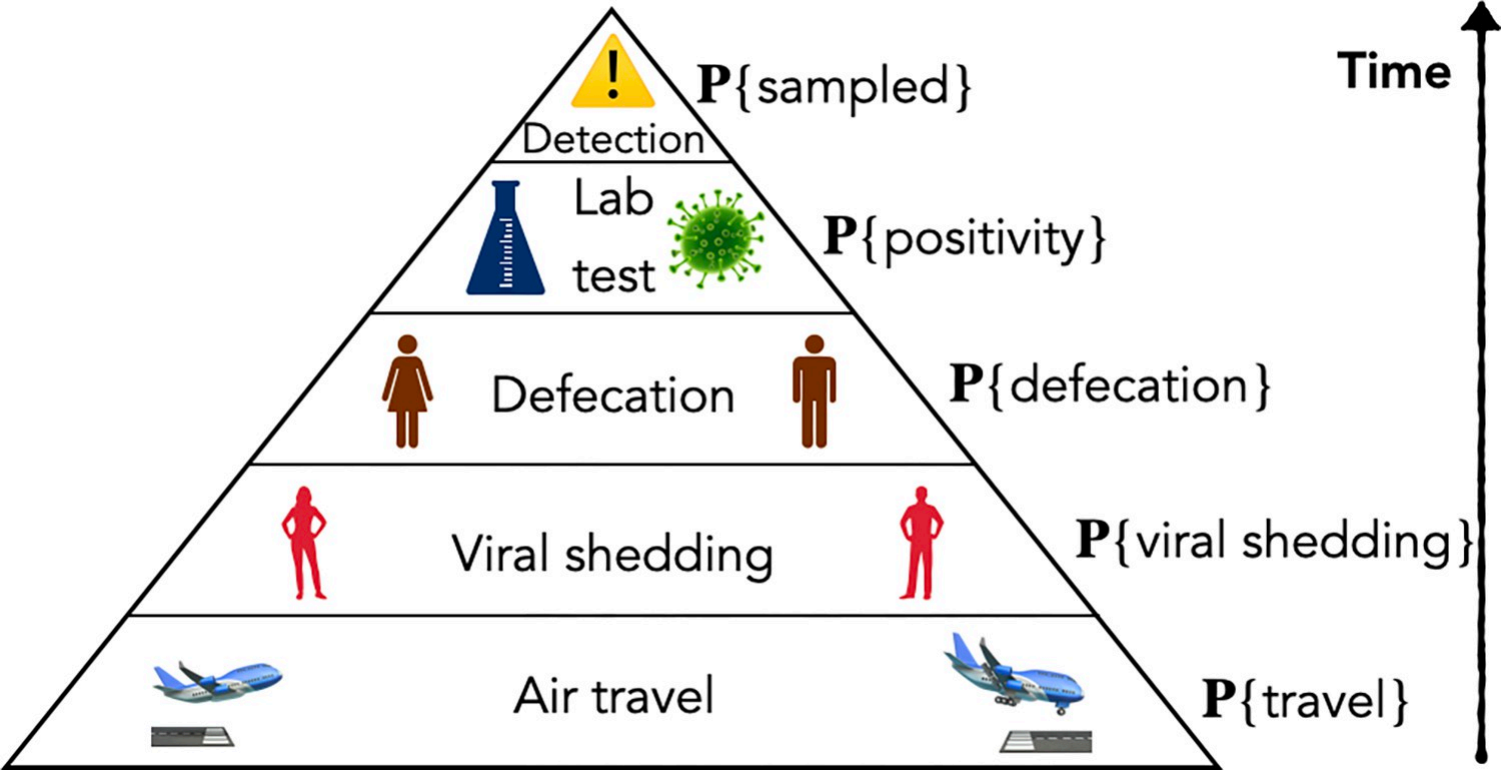
Model schematic. Graphical derivation of the final detection probability from the probabilities of air travel, viral shedding, defecation, fecal testing positivity, and sampling.

We calculated the probability of the virus being detected through aircraft wastewater surveillance by taking the product of the intermediate events necessary for a positive sample, illustrated in Fig 1 and described below.

### Inference of the actual infection size

The origin of SARS-CoV-2 has been traced back to zoonotic events in November 2019 [9, 10], before clusters of pneumonia cases caused by the virus began to emerge in late December [11]. The actual infection size in Wuhan was also much larger than observed in the first few months of the outbreak, as was demonstrated by previous modelling studies based on reported case counts, intensive care unit occupancy, and fatality rates [12, 13].

To incorporate these findings, we assumed an exponential growth in the number of infections that matched that of the initial days of the COVID-19 pandemic, and first fitted the logarithm of reported case counts in Wuhan from 15 January to 8 February 2020 [12] using linear regression. We assumed a constant ascertainment rate over that time period, and further estimated the actual infection size from the extrapolated and reported numbers of non-zoonotic cases between 8 December 2019 and 8 January 2020 [14], wherein a clinical detection rate of 20% was taken for the one-month period [15], under the assumption that only more clinically-severe SARS-CoV-2 infections were diagnosed during that time. With the estimated parameters, we then predicted the infection size on each day until 22 January 2020, one day before a lockdown was imposed in Wuhan by Chinese authorities [12].

### Proportion of viral shedding population

Let *I*_*t*_ be the inferred number of new infections on day *t*. Assuming the average duration of faecal viral shedding to be 20 days following infection (excluding the day of infection itself) [16, 17], the number of people shedding in this way on day *t* would be $\sum_{s=1}^{20}I_{t-s}$, and the probability of a stool sample being positive from a randomly selected individual in the epicentre (Wuhan) would be

$$p_{t}^{v}=P\{viral\;shedding\;on\;day\;t|Wuhanian\}=\sum_{s=1}^{20}I_{t-s}/N,$$

where *N* = 11 200 000 was the population in Wuhan by the end of 2019 [18].

### Probability of defaecation on board

Let $f_{def}(n),n\in \{0,1,2,3,4\}$, be the point mass function for the number of bowel movements per day (Table A, Fig A in S1 Text), derived from a recent study focusing on stool frequencies among healthy Singaporean residents. Additionally, let $f_{def}^{n}(t|n=1)$ denote the distribution of the defaecation time (*t* in minutes) during a day when an individual experiences only one bowel movement (Fig B in S1 Text), which was obtained from the relevant literature [19]. For people having *m*(>1) bowel movements in one day, we assumed equal time intervals between neighbouring defaecations, i.e.,

$$f_{def}^{n}(t|n=m)=\sum_{\left\{k:t+1440k/m\in [0,1440)\right\}}f_{def}^{n}(t+1440k/m|n=1).$$

Then, presuming a consistent timing of defecation across individuals of different genders, ages, and races, we calculated the probability of defaecation for an individual during the time interval [*T*_1_, *T*_2_] as

$$p_{\left[T_{1},T_{2}\right]}^{d}=P\{defecation\;in[T_{1},T_{2}]\}=\sum_{n=1}^{4}f_{def}(n)\cdot \min\left\{1,\int_{\left[T_{1},T_{2}\right]}f_{def}^{n}(s|n)ds\right\}.$$

Under the assumption that air travel and COVID-19 infection did not change the defaecation habits, the probability of defaecation for an infected individual with viral shedding during a flight from time *T*_1_ to *T*_2_ was

$$P\{defecation\;in[T_{1},T_{2}]|viral\;shedding\;on\;day\;t,traveler\}$$

$$=P\{defecation\;in[T_{1},T_{2}]\}=p_{\left[T_{1},T_{2}\right]}^{d}.$$

### Probability of positive wastewater samples from a plane

Assuming infections were concentrated in the epicentre, Wuhan, before the city was locked down on 23 January 2020, and all travelers resided Wuhan before their departure (i.e., there were no transit passengers),

P{Wuhanian|travel}=P{Wuhanian}=1

and

P{travel|Wuhanian}=P{travel,Wuhanian}=P{travel}.

If $\left[T_{jtm}^{1},T_{jtm}^{2}\right]$ was the scheduled time for the *m*-th direct flight (*m* = 1,2,⋯,*M*_*j*_) from Wuhan to airport *j* on day *t*, the probability for a randomly chosen passenger on board to defaecate would be

$$P\{defecation\;in[T_{jtm}^{1},T_{jtm}^{2}],viral\;shedding\;on\;day\;t|travel\}=P\{defecation\;in[T_{1},T_{2}]|viral\;shedding\;on\;day\;t,Wuhanian\}\times P\{viral\;shedding\;on\;day\;t|Wuhanian\}\times P\{Wuhanian|\;travel\}=p_{\left[T_{jtm}^{1},T_{jtm}^{2}\right]}^{d}p_{t}^{v}.$$

Given *p*_+_ = 0.5 as the average probability for the shedding of fecal SARS-CoV-2 RNA to be tested positive [17], the probability of producing a positive stool sample for the individual would then be

$$p_{jtm}^{+}=p_{+}p_{\left[T_{jtm}^{1},T_{jtm}^{2}\right]}^{d}p_{t}^{v}.$$

Let *v*_*jtm*_ be the number of passengers on broad, and the number of stool samples with viral shedding, $N_{jtm}∼Binom(v_{jtm},p_{jtm}^{+})$, under the assumption of equal chance of travelling for all the residents in Wuhan, as well as independence between people’s travel behaviour and infection. Therefore, the probability for the wastewater on the plane to test positive if tested was then

$$p_{jtm}^{f}=1-P(N_{jtm}=0)=1-{\left(1-p_{jtm}^{+}\right)}^{v_{jtm}}.$$

### Probability of detecting the virus at the airports

Provided that the authorities randomly sampled *τ* (∈(0,1]) of all the inbound flights to airport *j*, the probability of airport *j* reporting positive wastewater samples from Wuhan on day *t* would be

$$\phi_{jt}=1-\prod_{m=1}^{M_{j}}\left(1-\tau p_{jtm}^{f}\right),$$

and that of at least one airport in the subset *J* of all the airports included detecting positive wastewater samples on day *t* would be

$$\psi_{t}=1-\prod_{j\in J}\left(1-\phi_{jt}\right).$$

Hence, the probability that SARS-CoV-2 was detected from the aircraft wastewater by day *t* was

$$F(t):=P\{detected\;by\;day\;t\}=1-\prod_{s=t_{0}+1}^{t}\left(1-\psi_{s}\right),$$

and the probability that the virus was first detected on day *t* became

$$p(t)≔P\{first\;detected\;on\;day\;t\}=\psi_{t}\prod_{s=t_{0}+1}^{t-1}\left(1-\psi_{s}\right),$$

where *t*_0_ was the presumed start point of the pandemic.

#### Scenarios considered

We considered two hypothetical scenarios below:

1. Wastewater was tested from a proportion of all inbound flights to a single airport, operating its own surveillance system, with sampling probability *τ* = 1, 0.5, 0.2, and 0.1.
2. Wastewater was tested from a proportion of inbound flights to a collaborating network of 20 major airports in Asia, Europe, and North America, with sampling probability *τ* = 1, 0.5, 0.2, and 0.1.

**Fig 2:**
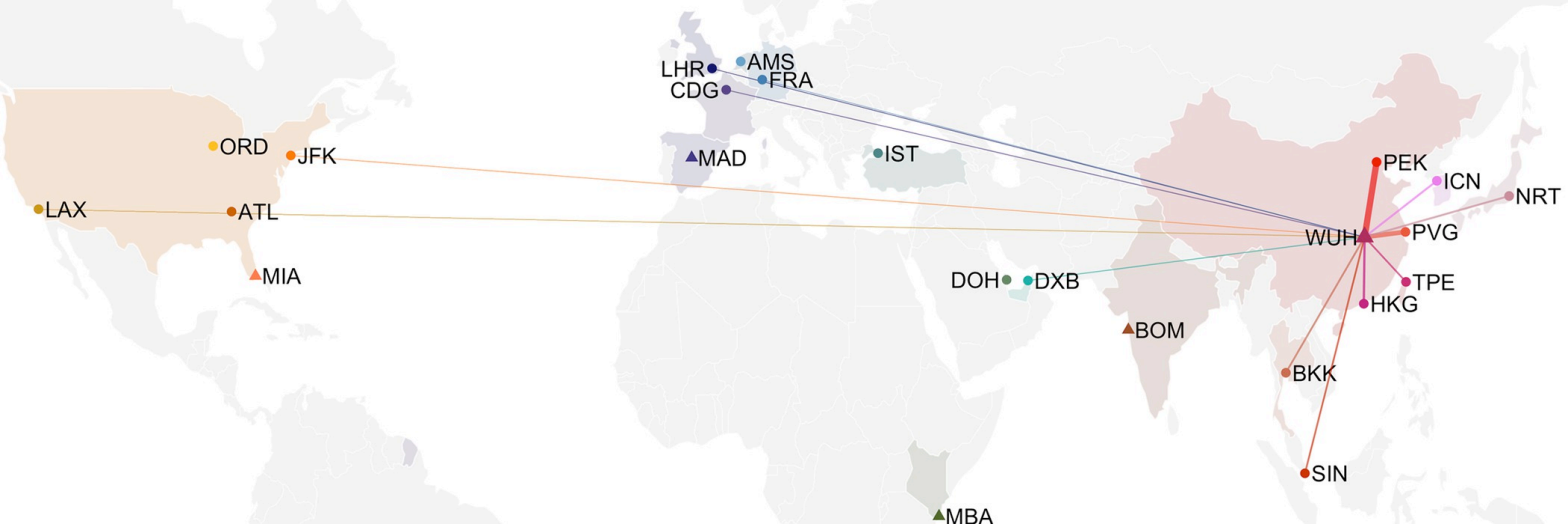
World map of the airports studied. Location of Wuhan (WUH), hypothetical epicentres (Madrid [Madrid], Miami [MIA], Mombasa [MBA], and Mumbai [BOM]), and a network of 20 major airports around the globe. The lines represent (postulated) direct flights from Wuhan in 2019, and the line widths are proportional to the (estimated) travel volume in 2019 [20].

In both cases, the scenarios were run until the point at which Wuhan was locked down on 23 January 2020, and the airports considered were NRT (Tokyo, Japan), ICN (Seoul, South Korea), TPE (Taipei, China), PVG (Shanghai, China), PEK (Beijing, China), HKG (Hong Kong SAR, China), SIN (Changi, Singapore), BKK (Bangkok, Thailand), DXB (Dubai, the UAE), DOH (Doha, Qatar), IST (Istanbul, Turkey), FRA (Frankfurt, Germany), AMS (Amsterdam, the Netherlands), CDG (Paris, France), LHR (London, the UK), MAD (Madrid, Spain), JFK (New York, the US), ATL (Atlanta, the US), ORD (Chicago, the US), and LAX (Los Angeles, the US) (Fig 2). These airports were selected according to international passenger traffic.

For these two scenarios, we postulated the flight information and estimated the number of passengers on board before the pandemic with 2017 air travel data [20] and the flight schedules in 2023 [21], assuming the growth in passenger volume from 2017 to 2019 was 18.5% [22], while for flights whose timetables are not available in 2023, we assumed they were equally likely to take off at any time of a day.

We additionally considered a future outbreak from any of the arbitrarily chosen alternative epicentres, comparable in growth to that in Wuhan in December 2019 and January 2020, which were: Madrid (MAD, Spain), Miami (MIA, the US), Mombasa (MBA, Kenya), Mumbai (BOM, India) (Fig 2). In this scenario, we also assumed routine aircraft wastewater surveillance would be performed in the aforementioned 20 major airports. Travel volume between different airports were derived based on the flight schedules in 2023 [21], while the number of passengers on board for each flight was taken to be around 85% of the seating capacity of the corresponding aircrafts (details in S1 Text).

## Results

**Fig 3:**
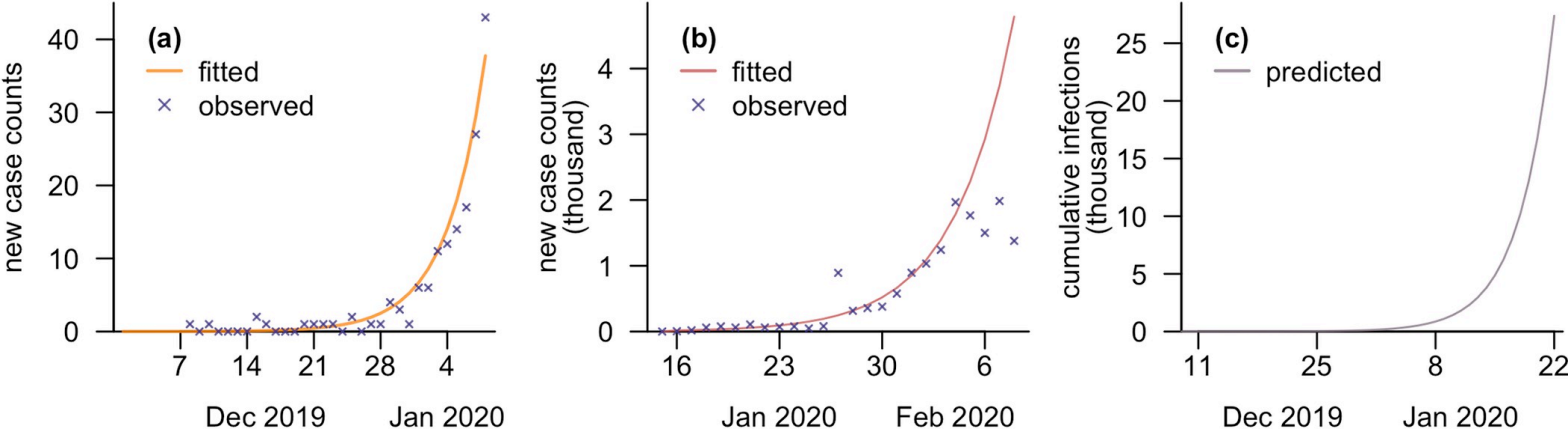
Validation and projection from the case model. (a) Fitted and observed number of new cases from 1 December 2019 to 8 January 2020, assuming only severe infections (20%) were detected; (b) fitted and observed number of new cases from 15 January to 8 February 2020, assuming a constant ascertainment rate; (c) predicted cumulative number of infections using our model, from 1 December 2019 to 22 January 2020, before Wuhan was locked down.

Fits of the case model and time series of estimated infection sizes are presented in Fig 3 for the early period of the COVID-19 pandemic. The start point of the pandemic was taken to be 9 December 2019, when the modelled cumulative case count first exceeded 0.5, and the growth rate was 0.28 per day. The cumulative number of infections as of 22 January 2020 was estimated to be over 27 000, with a daily increase of approximately 6000.

**Fig 4:**
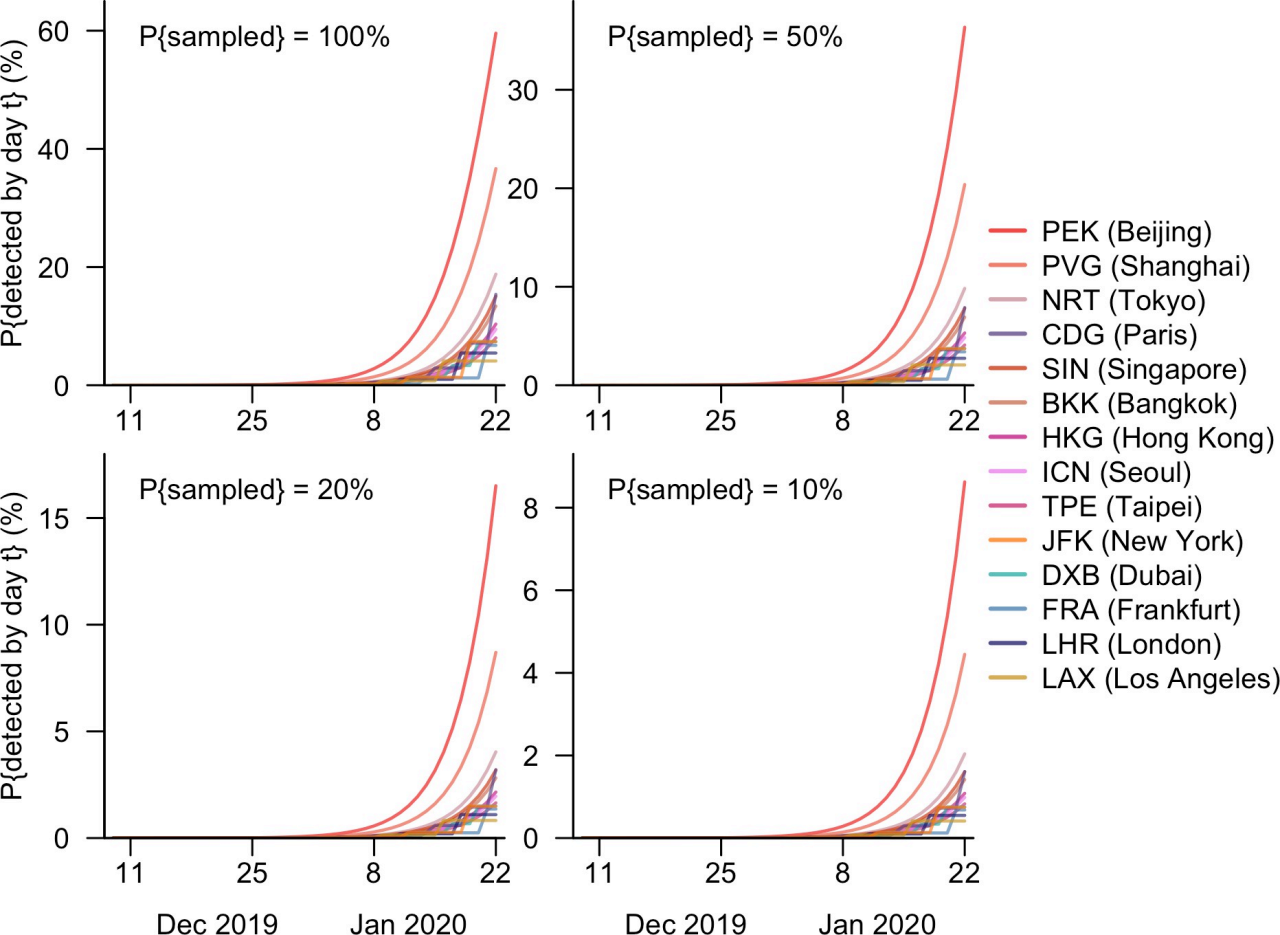
Cumulative probability of detecting SARS-CoV-2 against time in airports operating independently. The probability of SARS-CoV-2 having been detected by day *t* when the aircraft wastewater was sampled with a probability of 100%, 50%, 20%, or 10% in any one of the 14 airports with direct flights from Wuhan between 9 December 2019 and 22 January 2020.

Under the scenario in which each airport had and operated its own surveillance system, the detection probabilities would have been highest at Beijing Capital International Airport (PEK) across different sampling probabilities (Fig 4). Were wastewater testing performed on all inbound aircrafts at PEK, the probability of detecting SARS-CoV-2 by 22 January 2023 would have been 60%, 23 percentage points (pp) higher than that at Shanghai Pudong International Airport (PVG). Narita International Airport at Tokyo, which is approximately a five-hour flight from Wuhan and expected over 200 passengers from Wuhan every day before the pandemic, also would have had a reasonable chance of detection, higher than other airports outside mainland China. Despite the long distance to the epicentre and high number of defecation events on the plane, airports with flights from Wuhan only once a week (JFK, FRA, LHR, and LAX) would have been the least likely to detect viral spread in the early phase of the pandemic through aircraft wastewater testing (Fig 4, Table 1, Table D in S1 Text).

**Table 1:** Probabilities (%) of Beijing Capital International Airport (PEK) reporting positive wastewater samples from Wuhan on different days—30 December 2019, 7 January 2020, 13 January 2020, and 22 January 2020, assuming wastewater was tested from 100%, 50%, 20%, or 10% of inbound flights.

P{Sampled} (%)	30 Dec 2019	7 Jan 2020	13 Jan 2020	22 Jan 2020
**100**	0.31	2.20	9.3	59.6
**50**	0.15	1.11	4.8	36.6
**20**	0.06	0.44	1.9	16.5
**10**	0.03	0.22	1.0	8.6

**Fig 5:**
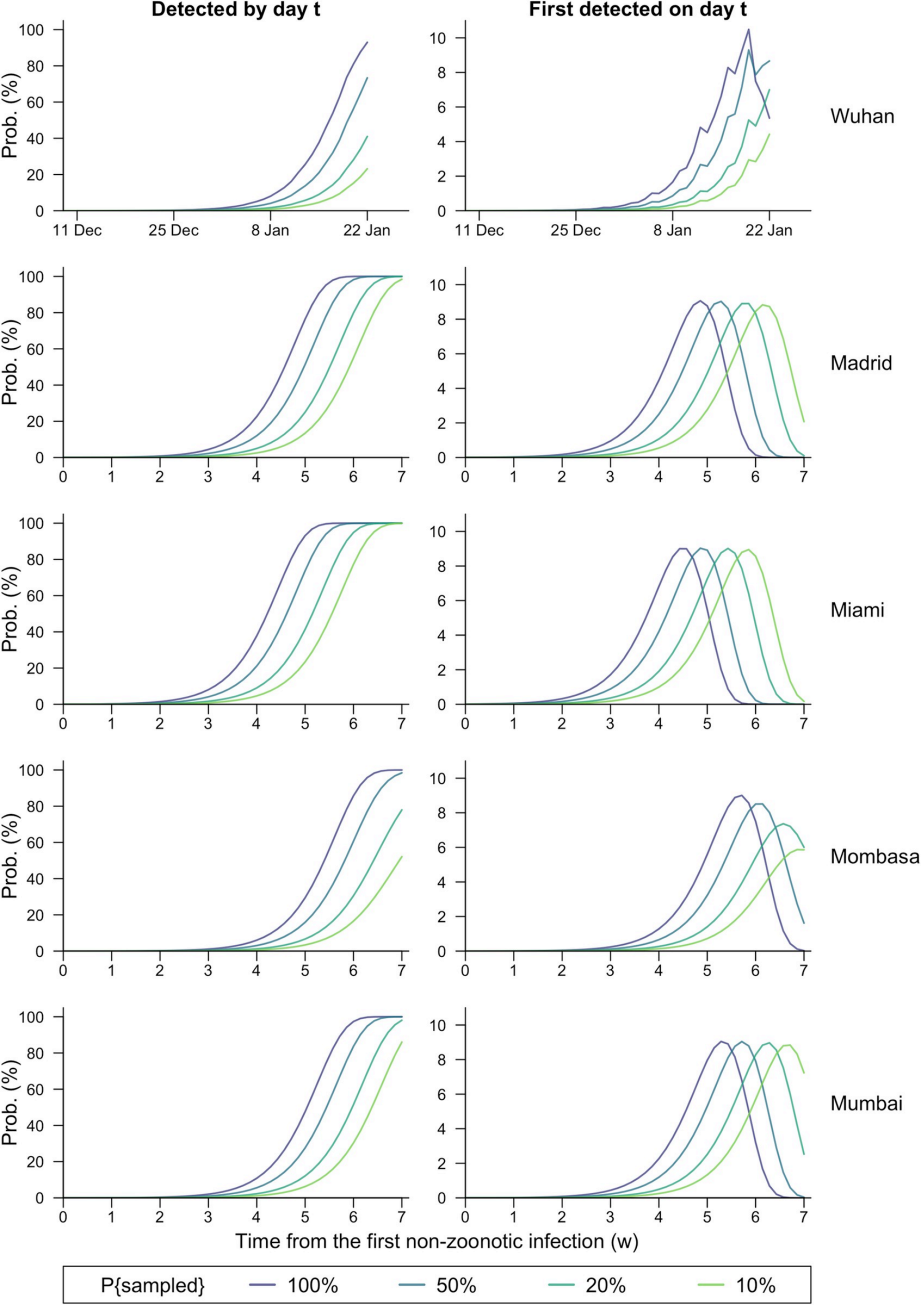
Detection probability against time for different epicentres. The probability of the virus having been detected by day *t* (column 1) or first detected on day *t* (column 2) under four scenarios with diverse sampling probabilities (100%, 50%, 20%, 10%) for inbound flights, assuming different epicentres—Wuhan, Madrid, Miami, Mombasa, and Mumbai—and routine aircraft wastewater surveillance at all the 20 airports investigated.

The probability of detecting the onset of the pandemic would, however, have been substantially increased if a network of 20 major airports in Asia, Europe and North America collaborated to test aircraft wastewater (Fig 5). If all inbound planes were sampled at those airports, the likelihood of the novel coronavirus having been detected by 22 January 2020 would have been as large as 93%, while modal time of detection would have been 19 January (Table 2). Even were the sampling probability reduced by half, the day when SARS-CoV-2 was the most likely to be detected would have remained the same, but the expected detection date would have been delayed. Nevertheless, in this scenario with a halved sampling probability, the chance of detecting the virus before Wuhan was locked down would have been higher (by 14pp) compared to the scenario which involved sampling all the inbound flights at PEK only, which was the most connected to Wuhan among the 20 airports investigated (Tables 1, 2).

**Table 2:** Probabilities (%) of the 20 major airports reporting positive wastewater samples from Wuhan by different time points—30 December 2019, 7 January 2020, 13 January 2020, and 22 January 2020, assuming wastewater was tested from 100%, 50%, 20%, or 10% of inbound flights.

P{Sampled} (%)	30 Dec 2019	7 Jan 2020	13 Jan 2020	22 Jan 2020
**100**	0.92	6.37	25.5	93.0
**50**	0.46	3.24	13.7	73.4
**20**	0.18	1.31	5.7	41.0
**10**	0.09	0.66	2.9	23.2

Being loosely proportional to infection size, the detection probability would have also increased exponentially in the early phase of the COVID-19 outbreak in Wuhan. Even in the optimal scenario when all inbound flights were under wastewater surveillance at the 20 major airports, the chance of detecting the novel virus was merely 0.9% by 30 December 2019, when several pneumonia cases of unknown origin were reported to China National Health Commission [11], but the likelihood quickly rose to 6.4% in eight days and further exceeded 25% in another week’s time (Table 2). It was also worth noting the particularly prominent difference in detection probabilities between scenarios involving only one and all airports in December 2019 and early January 2020. For instance, compared to operating on an airport’s own surveillance, the collaboration between the 20 airports would have at least doubled chance of detecting the virus through aircraft wastewater surveillance before COVID-19 cases started to be clinically diagnosed in cities outside mainland China on 13 January 2020 (Table 2).

In addition, a non-linear decrease was found in the probability of detecting SARS-CoV-2 through aircraft wastewater testing to the sampling proportion, *τ*, of inbound planes (S3 Fig). Specifically, the reduction in detection probability for any specific day caused by decreasing *τ* would have been larger in scenarios with smaller *τ*s, while increasing *τ* would have led to a smaller relative increase in detection probabilities against time. Furthermore, changes in sampling proportion within certain ranges might not postpone the modal time of detection (Fig 5, Fig D in S1 Text).

We also considered hypothetical outbreaks emerging outside China (in Madrid, Miami, Mombasa, and Mumbai). Among the four pseudo-outbreaks, if all airports sampled the same proportion of inbound aircrafts’ wastewater, the outbreak emerging around Miami, US, would be most likely to be detected early. If wastewater from all inbound flights were tested at the 20 airports, the virus would most likely be detected 31 days after the day with the first non-zoonotic infection (Fig 5, Table 3), and the probability for viral detection between the 19^th^ and the 38^th^ day exceeded 95%. This was followed by the outbreak with Madrid as the epicenter, which would take a further three days to detect. Both cities are smaller than Wuhan but have frequent direct flights to major airports in the US, Europe, and the Middle East (Tables C, E, F in S1 Text). In contrast, outbreaks originating in Mumbai and Mombasa would take longer to detect (by 6 and 9 days, respectively, compared to Miami). The modal detection days, nevertheless, were within two weeks, suggesting that a novel pandemic with similar characteristics to COVID-19 has a reasonable chance to be detected at an early stage of its development by such a network, regardless of the continent it emerged from (Table 3).

**Table 3:** The day with the highest probability of the virus being first detected ($p(\cdot ))(i.e.,t_{max}=\arg\underset{t}{\max}p(t)$, i.e., the mode), cumulative (*F*(⋅)) and new (*p*(⋅)) detection probabilities by day *t*_*max*_, and the 95% highest density (time) interval (HDI) for virus detection for Wuhan and the other four hypothetical epicentres, assuming the day with the first non-zoonotic infection is day 1 and wastewater from all the inbound aircrafts to the 20 major airports are tested.

Epicentre	*t*_*max*_	*p*(*t*_*max*_) (%)	*F*(*t*_*max*_) (%)	95% HDI for detection
Wuhan*	42	10.5	73.5	[1,45]
Madrid	35	9.1	67.6	[19,40]
Miami	32	9.0	63.1	[19,38]
Mombasa	41	9.0	69.7	[27,47]
Mumbai	38	9.0	65.3	[24,44]

* Time series of modelled infection sizes and detection probabilities for Wuhan were truncated by 22 January 2020 (day 45), since people were banned from leaving the city except for special reasons since the city was locked down on 23 January 2020. The cumulative detection probability by day 45 for Wuhan was 0.93.

## Discussion

This study aims to evaluate the potential utility of aircraft wastewater testing for the detection of a novel pathogen, using the initial outbreak of SARS-CoV-2 in Wuhan, China as a proof-of-concept example. Although no airport was recorded to have performed routine monitoring before or at the start of the COVID-19 outbreak, aircraft wastewater surveillance has begun to be utilized to detect the presence of SARS-CoV-2 since the early phase of the pandemic [23–26], albeit after the pandemic was known. Despite its wide use and effectiveness [23–26], there are several conditions requisite for a positive test result, including the presence of one or more infected and shedding travelers, that they defaecate on board, that wastewater from the plane be collected and tested, and that the concentration of the virus reach the threshold to be detected by test equipment. Quantifying likelihoods of these events enables us to theoretically assess the potential of identifying the emerging virus before its existence was known, in the hypothetical scenario in which wastewater-based epidemiology was routinely practised on inbound aircrafts at major airports around the world.

It should also be noted that surveillance at the epicentre itself is more effective than traveller surveillance, as showed by the much earlier detection and reporting of the COVID-19 outbreak in Wuhan compared to the very low probability of detecting the virus at the same date in any of the modelled scenarios in our study, due to the good surveillance and reporting systems in place in China. However, this cannot be assumed for many other cities across the world, and therefore traveller surveillance is a potential solution for early detection of novel disease outbreaks, compared to population-level surveillance which often necessitates the outbreak spreading and becoming entrenched across the globe.

We found in our analysis that while it may not be feasible to rely on a single airport to identify the virus from aircraft wastewater through its own monitoring system, likelihoods of detection would be substantially increased if a network of major airports cooperated to test wastewater from inbound flights, either in a scenario matching the emergence of COVID-19 in Wuhan, or in scenarios for epicentres in other continents. However, it should also be noted that our estimations did not show a prominent improvement in the time of detection by aircraft wastewater surveillance compared with other methods, such as the testing of individual travelers, but its lower burden in both costs and implications increases the competence of the proposed approach [27, 28].

Our findings underscore the potential advantages for aircraft wastewater surveillance as a complementary approach for viral detection, particularly in regions with limited access to disease surveillance information due to the absence of robust reporting systems. In addition, these results highlight the significance of global cooperation among airports in conducting aircraft wastewater testing to enhance the prospects of timely detection, facilitate information sharing among health authorities, and expedite introduction of timely interventions to effectively curb the spread of diseases. Such collaborative surveillance networks could be guided by international guidelines, including those outlined in the International Health Regulations [29], to ensure a coordinated and standardized approach to cross-border surveillance.

We further evaluated the impact of reduction in sampling probability on testing performance. We observed a decrease in the marginal benefits with the increase in the chance of an aircraft being sampled, suggesting selective testing as a viable strategy to alleviate the surveillance burden while maintaining effectiveness.

To lend the analysis more fidelity, we innovatively incorporated flight arrangements—including flight duration, the exact departure time in a day and days with scheduled flights in a week—in the calculations and considered the imbalanced time of day distribution for bowel movements. A sensitivity analysis indicated the exclusion of these heterogeneities in flight schedules might affect the inference results to some extent, but not fundamentally, since major contributors to detection were either short, frequent flights taking off at multiple times in a day, or cross-continental flights with over 10 hours on the plane, both of which were barely constrained by timings.

There are, however, limitations in our study worth highlighting. The possible existence of stochasticity in the actual infection size would not be captured by the exponential model of case counts used in our inference, but we showed in a sensitivity analysis its negligible impact on the eventual calculation results (Fig A in S3 Text). We did not account for the delay in collecting and testing wastewater samples, nor did we gauge the potential variation in testing sensitivity at different airports [17]. The probability of defaecating on board, a crucial determinant of the eventual viral detection, could be affected by people’s inclination to evacuate pre-departure or post-arrival, especially for short-haul flights with only a few hours on board, as was suggested by Jones *et al* and *Shingleton et al*. [8, 30]. Results of the survey in Jones *et al*’s work [8], nonetheless, failed to demonstrate a significant reduction in the respondents’ willingness to use toilets. This is an understudied area, however, and data on the relationship between defaecation rates and flight durations are scarce. Sensitivity analysis showed the chance of detecting the virus would be greatly reduced if proclivity to defaecate in the air were lower than on land (Fig B in S3 Text), slowing the time until detection (Table A in S3 Text). Furthermore, toileting habits are subject to people’s diets and vary among different races [31]. In the absence of pertinent studies, we adopted data on the timing of defaecation from a study involving healthy American males. Nevertheless, it should be emphasized that we relied on Singaporean data regarding the frequency of passing motion (Table A in S1 Text). Although the potential disparity between data from these two distinct populations could introduce bias to our analysis, it is worth noting that we did not find sufficient evidence in existing literature to support the idea that the time-of-day distribution of defecaecation varied greatly by gender, age, or race. This suggests that our use of Singaporean data is likely a reasonable proxy for travelers originating from Wuhan, which serves as the baseline setting for our models, but may not extrapolate to other settings.

The detection probabilities might also be underestimated due to various reasons, including alternative channels—such as vomit and urine—for the virus to enter wastewater, and increased toileting activities due to diarrhoea caused by infection. Such incidents have proven to be less common for COVID-19, but could be more common for other diseases [2, 32]. A stronger influence, by comparison, would be the risk that infected individuals transited via air hubs distant from the epicentre, which would inflate the chance of identifying the virus at foreign destinations and bring forward the modal time of detection, but this may bring additional challenges to discerning the exact location of the outbreak.

Despite these limitations, taking the outbreak of COVID-19 in Wuhan as an example, our study provides insights into the potential of identifying the source of an emerging pathogen through collaborative aircraft wastewater surveillance, employing a probabilistic approach. As most countries have transitioned to a new normal of living with COVID-19 and lifted border measures such as travel restrictions and screening requirements, cross-border travel is gradually resuming and even exceeding the pre-pandemic levels [33]. Our analysis lays the theoretical foundation for the prospective application of aircraft wastewater monitoring to collaborative epidemiological surveillance at multiple national borders, by identifying the presence and assessing the potential spread of new SARS-CoV-2 variants of concern, or another unknown disease X with high rates of viral shedding in wastewater.

## Supporting information

S1 Text Appendix containing additional details on data and methodology employed in this study. Further information on how the actual infection size and defecation frequency were derived, IRB approval for the defaecation data, along with data utilised for probability calculation.
(DOCX)

S2 Text Appendix containing additional details on results for the main analysis. Further information on the derived detection probabilities and delay in modal detection times due to subsampling in the main analysis.
(DOCX)

S3 Text Appendix containing sensitivity analyses. A list of sensitivity analyses performed for alternative sets of parameters or scenarios considered for probability calculation.
(DOCX)
