# Supplementary material for: Feasibility of wastewater-based detection of emergent pandemics through a global network of airports
Source: PLOS Glob Public Health. 2024 Mar 13;4(3):e0003010. doi: 10.1371/journal.pgph.0003010 (PMC10936834; doi:10.1371/journal.pgph.0003010)
Supplement: S1 Text — Further information on how the actual infection size and defecation frequency were derived, IRB approval for the defaecation data, along with data utilised for probability calculation. (DOCX) [file pgph.0003010.s001.docx]

**S1 Text. Appendix containing additional details on data and methodology employed in this study**

**The actual infection size**

Let $L_{t}$ be the logarithm of reported cumulative case counts in Wuhan from 15 January to 8 February 2020 [1]. We considered the following linear regression model:

$L_{t}=\alpha+\beta t+\epsilon_{t}, \epsilon_{t}\sim N\left( 0,\sigma^{2} \right) i.i.d.$,

where $\beta$ is the slope and $e^{\beta}$ is the growth rate. We fitted the data and obtained the maximum likelihood estimator (MLE) of $\beta$ to be 0.247, equivalent to a growth rate of 0.28 per day.

Assuming a constant ascertainment rate, $r$, over that period, the model we constructed for $r$ was

$E_{t}\sim\mathrm{Poisson}(sr^{-1}\exp\left( \hat{L}_{t} \right))$,

where $E_{t}$ was the cumulative number of clinically diagnosed non-zoonotic cases between 8 December 2019 and 8 January 2020 [2], $s=0.2$ was the proportion of severe cases in this early phase of the outbreak [3], and $\hat{L}_{t}$ was the projected log case counts during this period from the previous linear regression model.

The MLE for $r$ was 0.012, from which we obtained the estimated (cumulative) infection size at time $t$, $C_{t}$, as

$C_{t}=\hat{r}^{-1}\exp(\hat{L}_{t})=\hat{r}^{-1}\exp(\hat{\alpha}+\hat{\beta}t)$,

where $\hat{r}$, $\hat{\alpha}$ and $\hat{\beta}$ were the MLEs, and $t$ was the time variable, ranging from 1 December 2019 to 22 January 2020. For scenarios of pseudo-outbreaks, we ran the estimates until day 50 of the outbreaks (i.e., estimated $C_{t}$s for a range of 58 days). The number of new infections on each day from 9 December 2019 (the start of the epidemic) to 22 January 2020 would then be

$I_{t}=C_{t}-C_{t-1}$.

**Defaecation frequency**

Distribution for number of defaecations per day (Table A) was derived from the self-reported stool frequencies of 1307 mostly healthy individuals in Singapore (Fig A), among whom 83% had Chinese parents. Other major races in the sample included Indian (7%) and Malay (3%). Additional demographic information of these individuals is provided in Table B. These participants were involved in a microbiome study by AMILI Pte Ltd and had given their informed consent for their data to be used for research (NUS IRB 2020/0501).

Table A. Distribution for number of defaecations per day calculated from self-reported stool frequencies in Singapore (Fig A).

| **Times/day** | **0** | **1** | **2** | **3** | **4** |
| --- | --- | --- | --- | --- | --- |
| **Proportion (%)** | 13.8 | 69.1 | 15.5 | 1.1 | 0.5 |


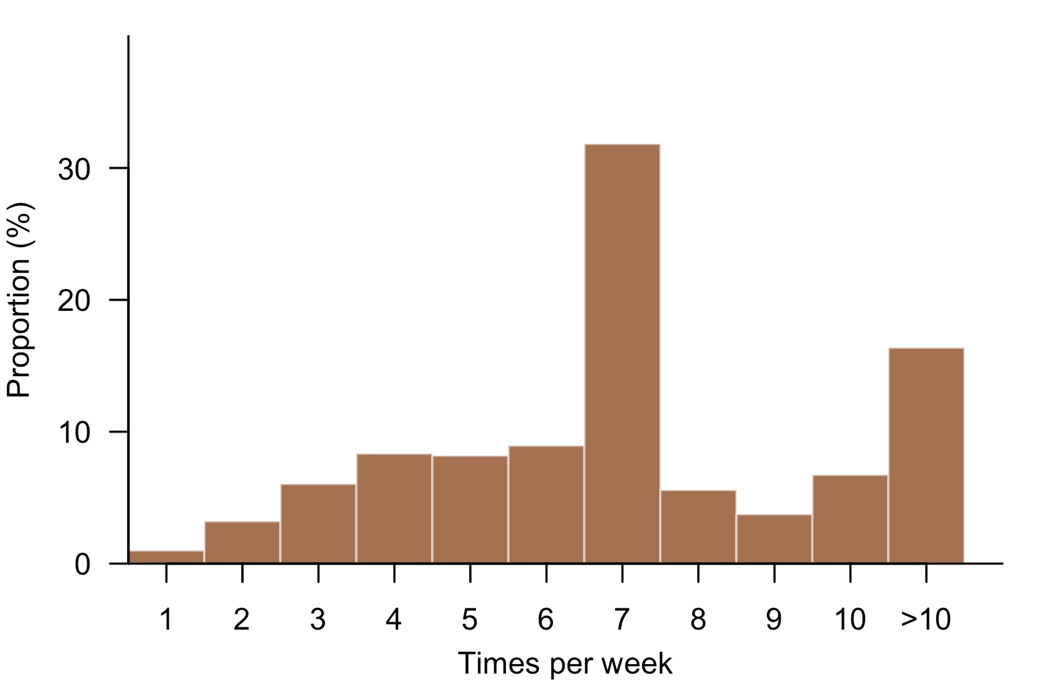


Fig A. Proportions of the 1307 individuals with different defaecation frequencies.

Table B. Demographic information of the 1307 individuals involved in the study of stool frequencies (mean and standard deviation for age, counts and proportions for gender and parental race).

| **Age** | 43 (14) |
| --- | --- |
| **Gender** |  |
| Female | 773 (59%) |
| Male | 532 (41%) |
| **Parental race** |  |
| Chinese | 1082 (83%) |
| Indian | 96 (7%) |
| Malay | 35 (3%) |
| Others | 92 (7%) |

For individual $i$reporting $k^{i}=7k_{1}^{i}+k_{2}^{i}(k_{1}^{i}-1)$ (both $k_{1}^{i}$ and $k_{2}^{i}$ are non-negative integers) times per week, we assumed in one day he would defaecate $(k_{1}^{i}-1)$ times with probability $k_{2}^{i}/7$ and $k_{1}^{i}$ times with probability $(1-k_{2}^{i}/7)$. Then, the point mass function for defaecation frequency per day is

$f_{def}\left( n \right)=\sum_{i} \boldsymbol{1}_{k_{1}^{i}=n}\cdot k_{2}^{i}/7+\sum_{i} \boldsymbol{1}_{k_{1}^{i}=n+1}\cdot(1-k_{2}^{i}/7)$.

Since the maximum frequency per week in the raw data was truncated at 11, we also refer to related statistics in literature[4,5] for proportions of people defaecating three times or more per day. More specifically, we followed Chen *et al.*’s work [4], assigning 0.5% to the probability of defaecating four times a day, and slightly inflated the rate of three defaecations per day in the study by Fang *et al* [5]*.* to allow the possibility of people who normally defaecate twice a day to occasionally have another bowel movement.

Time of day distribution for defaecation given a defaecation frequency of one per day, $f_{def}^{n}\left( t \right| n=1)$, was approximated from the empirical distribution of 8267 samples[6] (Fig B) using ‘gam’ function in the R package *mgcv* [7], whence a point mass was assigned to every minute of a day.


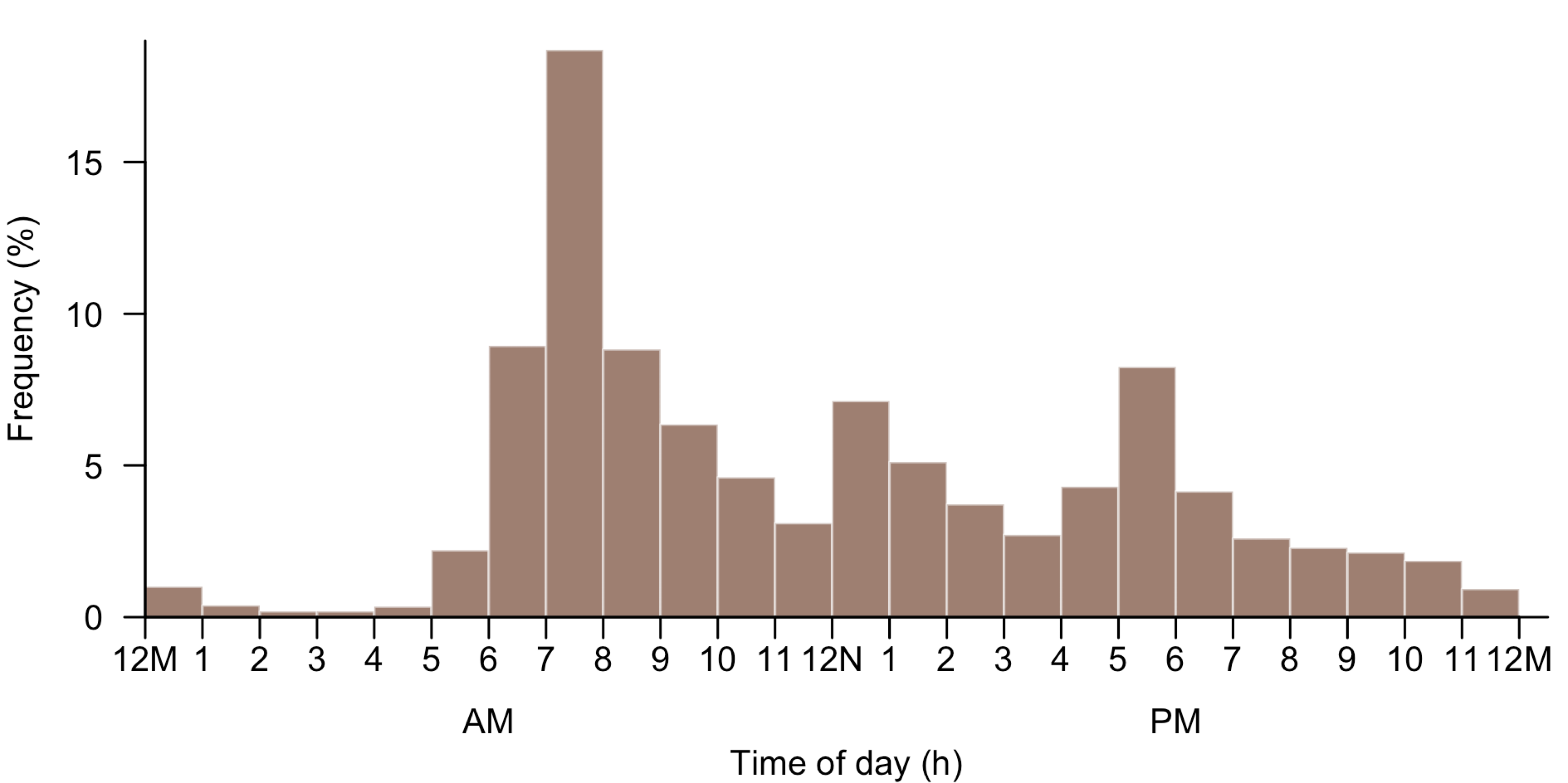


Fig B. Histogram of defaecation time for the 8267 samples in literature [6].

**IRB approval for defaecation frequency data (Singapore) and brief notes about source of participants**

Singapore citizens or permanent residents and long-term residents, aged 21 years old and above, were recruited into AMILI’s Microbiome Donor Programme and completed their responses via online survey platform hosted on AMILI’s website from May 2020 – February 2023 with informed consent. The programme is an ongoing project funded by AMILI Pte Ltd with ethics approval obtained under AMILI institutional review board.

Subset of the questions asked from the programme was extracted and incorporated into the model. Briefly, a total of 1313 responses were obtained and paired with demographics information on age, gender, BMI, ethnicity, age and bowel habits such as frequency of bowel movement per day and per week, stool Bristol score, use of products with the sole purpose of changing or influencing bowel movement for defaecation, presence of blood in stool and bowel difficulty.

**Data used for probability calculation**

Table C. Population of Wuhan[8] (2019) and the four hypothetical epicentres[9] (2023).

| **City** | **Wuhan**  **(China)** | **Madrid**  **(Spain)** | **Miami**  **(the US)** | **Mombasa**  **(Kenya)** | **Mumbai**  **(India)** |
| --- | --- | --- | --- | --- | --- |
| **Population**  **(million)** | 11.2 | 6.7 | 6.3 | 1.44 | 21.3 |

Table D. Postulated flight schedules for outbound flights from Wuhan (WUH) and the numbers of passengers on board in December 2019 and 2020.

| **Destination**  **airport** | **City** | **Frequency** | **Passengers**  **(per day)** | **Duration** | **Departure time**  **(time of day)** |
| --- | --- | --- | --- | --- | --- |
| **NRT** | Tokyo | 1/d | 217 | 4h55min | 9:35 |
| **ICN** | Seoul | 1/d | 230 | 2h50min | 14:00 |
| **TPE** | Taipei | 1/d | 177 | 2h35min | 18:50 |
| **PVG** | Shanghai | 8/d | 1144 | 1h50min | 8:00; 9:30; 10:00; 15:00; 17:00; 18:00; 19:00; 19:30 |
| **PEK** | Beijing | 13/d | 2094 | 2h10min | 8:00; 9:00; 10:30; 11:00; 12:00; 13:30;14:30; 16:00; 16:30; 18:00; 19:30; 20:30;22:00 |
| **HKG** | Hong Kong | 2/d | 292 | 2h15min | 11:35; 20:50 |
| **SIN** | Singapore | 1/d | 180 | 5h | 12:00 |
| **BKK** | Bangkok | 1/d | 196 | 3h50min | 11:00 |
| **DXB*** | Dubai | 2/w | 57 | 7h40min | Every Monday  and Friday |
| **FRA*** | Frankfurt | 1/w | 63 | 10h30min | Every Tuesday |
| **CDG*** | Paris | 2/w | 28 | 11h30min | Every Wednesday  and Sunday |
| **LHR*** | London | 1/w | 39 | 11h | Every Saturday |
| **JFK*** | New York | 1/w | 32 | 15h30min | Every Sunday |
| **LAX*** | Los Angeles | 1/w | 40 | 14h | Every Thursday |

*Since there were no direct flight from Wuhan to these airports as of March 2023, we arbitrarily assigned days in a week with flights according to the travel volume, but assumed these flights were equally likely to take off at any time of a day (i.e., probability of defaecation on board was averaged over all the possible departure time points).

.

Table E. Postulated flight schedules for outbound flights from Madrid (MAD) and the numbers of passengers on board in 2023.

| **Destination**  **airport** | **City** | **Frequency** | **Passengers**  **(per day)** | **Duration** | **Departure time**  **(time of day)** |
| --- | --- | --- | --- | --- | --- |
| **ICN** | Seoul | 4/w | 121 | 13h35min | 20:00 |
| **HKG** | Hong Kong | 5/w | 213 | 12h40min | 12:20 |
| **DXB** | Dubai | 2/d | 850 | 7h25min | 17:18; 22:05 |
| **DOH** | Doha | 2/d | 510 | 6h45min | 9:25; 16:10 |
| **IST** | Istanbul | 3/d | 484 | 4h15min | 11:55; 14:25; 18:00 |
| **FRA** | Frankfurt | 9/d | 1128 | 2h50min | 8:40; 16:05; 19:55; 6:15; 8:30; 12:55; 17:05; 7:10; 15:10 |
| **CDG** | Paris | 9/d | 1224 | 2h20min | 6:00; 10:05; 12:40; 16:40; 17:50; 20:15; 8:45; 19:55; 20:40 |
| **LHR** | London | 13/d | 1920 | 2h35min | 10:45; 12:10; 22:07; 21:18; 21:00; 6:50; 7:40; 9:00; 13:20; 14:45; 15:50; 16:40; 17:15 |
| **JFK** | New York | 3–4/d | 956 | 9h45min | 10:45; 10:45; 12:30; 16:25 |
| **ATL** | Atlanta | 1/d | 208 | 10h45min | 11:00 |
| **ORD** | Chicago | 1/d | 212 | 10h55min | 11:35 |
| **LAX** | Los Angeles | 4/w | 170 | 13h45min | 12:25 |

Table F. Postulated flight schedules for outbound flights from Miami (MIA) and the numbers of passengers on board in 2023.

| **Destination**  **airport** | **City** | **Frequency** | **Passengers**  **(per day)** | **Duration** | **Departure time**  **(time of day)** |
| --- | --- | --- | --- | --- | --- |
| **DXB** | Dubai | 1/d | 280 | 14h15min | 21:10 |
| **DOH** | Doha | 1/d | 280 | 14h15min | 21:15 |
| **IST** | Istanbul | 1–2/d | 401 | 11h25min | 11:20; 22:05 |
| **FRA** | Frankfurt | 1/d | 340 | 9h10min | 16:45 |
| **AMS** | Amsterdam | 3/w | 91 | 8h40min | 17:50 |
| **CDG** | Paris | 2–3/d | 674 | 9h | 20:15; 20:50; 23:35 |
| **LHR** | London | 5–6/d | 1470 | 9h25min | 0:05; 21:00; 17:00; 22:30; 16:25; 20:10 |
| **MAD** | Madrid | 4/d | 916 | 9h | 18:05; 17:05; 22:55; 21:30 |
| **JFK** | New York | 19/d | 2728 | 3h20min | 7:05; 9:00; 10:30; 12:15; 13:30; 15:30; 17:30; 19:30; 20:30; 21:30; 7:45; 11:35; 15:20; 19:15; 7:05; 10:55; 15:00; 17:10; 20:55 |
| **ATL** | Atlanta | 19–20/d | 3283 | 2h15min | 8:30; 13:40; 15:30; 19:30; 21:00; 6:00; 7:00; 8:20; 10:15; 12:00; 14:05; 16:45; 18:00; 19:30; 17:00; 7:50; 17:00; 6:20; 23:50; 16:25; 6:20; 5:45; 16:55 |
| **ORD** | Chicago | 13/d | 1844 | 3h40min | 6:50; 8:30; 11:20; 13:40; 15:25; 17:30; 19:30; 21:00; 7:25; 12:15; 16:15; 12:12; 14:00 |
| **LAX** | Los Angeles | 11/d | 1716 | 6h6min | 6:10; 8:30; 11:15; 13:40; 15:30; 19:18; 19:30; 21:00; 8:15; 6:40; 19:50 |

Table G. Postulated flight schedules for outbound flights from Mombasa (MBA) and the numbers of passengers on board in 2023.

| **Destination**  **airport** | **City** | **Frequency** | **Passengers**  **(per day)** | **Duration** | **Departure time**  **(time of day)** |
| --- | --- | --- | --- | --- | --- |
| **DXB** | Dubai | 4/w | 78 | 5h35min | 23:10 |
| **IST** | Istanbul | 4/w | 78 | 7h10min | 4:40 |
| **FRA** | Frankfurt | 4/w | 121 | 9h10min | 11:00 |

Table H. Postulated flight schedules for outbound flights from Mumbai (BOM) and the numbers of passengers on board in 2023.

| **Destination**  **airport** | **City** | **Frequency** | **Passengers**  **(per day)** | **Duration** | **Departure time**  **(time of day)** |
| --- | --- | --- | --- | --- | --- |
| **NRT** | Tokyo | 1/d | 212 | 9h5min | 19:40 |
| **HKG** | Hong Kong | 1/d | 280 | 5h55min | 1:45 |
| **SIN** | Singapore | 4–5/d | 1080 | 5h55min | 23:50; 11:50; 1:00; 23:40; 0:55 |
| **BKK** | Bangkok | 5–6/d | 982 | 4h40min | 1:40; 2:55; 23:20; 8:50; 12:45; 7:50 |
| **DXB** | Dubai | 16–17/d | 3169 | 3h35min | 2:35; 8:15; 15:30; 19:35; 1:05; 8:25; 20:20; 4:30; 10:10; 15:35; 19:20; 22:20; 5:20; 15:15; 1:50; 23:05; 16:25 |
| **DOH** | Doha | 4–5/d | 766 | 4h10min | 4:55; 17:50; 7:45; 4:25; 18:20 |
| **IST** | Istanbul | 1/d | 280 | 7h10min | 6:35 |
| **FRA** | Frankfurt | 1/d | 230 | 9h25min | 2:55 |
| **AMS** | Amsterdam | 1/d | 212 | 9h35min | 2:25 |
| **CDG** | Paris | 1/d | 298 | 9h50min | 2:05 |
| **LHR** | London | 7/d | 1706 | 10h30min | 5:15; 14:00; 1:55; 8:50; 13:10; 10:40; 4:45 |
| **JFK** | New York | 1/d | 280 | 7h | 0:55 |

Table I. Passenger capacity and estimated actual load (85% of the capacity) for different types of aircrafts.

| **Aircraft (code)** | **Passenger capacity (n)** | **Actual load (n)** |
| --- | --- | --- |
| **A320** | 160 | 136 |
| **A330** | 250 | 213 |
| **A340** | 270 | 230 |
| **A350** | 350 | 298 |
| **A380** | 500 | 425 |
| **B737** | 160 | 136 |
| **B747** | 400 | 340 |
| **B757** | 240 | 204 |
| **B767** | 245 | 208 |
| **B777** | 330 | 281 |
| **B787** | 250 | 213 |
| **CRJ1000** | 104 | 88 |

**Averaging over different starting days of a pseudo outbreak**

Since flights between two airports may not be on a daily basis, the detection probabilities can be subjected to the starting day of outbreak in a week. As a pseudo outbreak is equally likely to take begin on any day of a week, for the scenario with hypothetical epicentres (i.e., Madrid, Miami, Mombasa, and Mumbai), we calculated the detection probabilities by averaging over all days of the week. That is, we derived the likelihoods assuming the pseudo outbreak started on each day of a week in turn, after which we took the mean of the 7 probabilities for each time point $t$ (time from the outbreak) as our final estimate.

**Summary of the key parameters in the models**

Table J. Explanations and data sources for some key parameters in the models.

| **Notation** | **Explanation** | **Values and source** |
| --- | --- | --- |
| $I_{t}$ | Inferred number of infections on day $t$ | Fig 3 [1–3] |
| $f_{def}(n)$ | Distribution for the number of bowel movements each day | Table A, from a microbiome study by AMILI Pte Ltd |
| $f_{def}^{n}\left( t \right\vert n)$ | Distribution for the defaecation time, $t$, when an individual has $n$bowel movements in one day | Fig B [6] |
| $[T_{1}, T_{2}]$ | Time interval for a flight, starting at $T_{1}$ and ending at $T_{2}$ | Table D–H [10,11] |
| $v_{jtm}$ | Number of passengers on broad | Table I (Postulated) |
| $N$ | Population of the epicentre | Table C [8,9] |
| $\tau$ | Sampling probability for inbound flights | $\{1, 0.5, 0.2, 0.1\}$ (Postulated) |
| $p_{+}$ | The average probability for the shedding of faecal SARS-CoV-2 RNA to be tested positive | 0.5 [12] |

**References**

1. Joseph T. Wu, Kathy Leung, Mary Bushman, Nishant Kishore, Rene Niehus, Pablo M. de Salazar, et al. Estimating clinical severity of COVID-19 from the transmission dynamics in Wuhan, China. Nat Med. 2020;26: 506–510. doi:https://doi.org/10.1038/s41591-020-0822-7

2. Li Q, Guan X, Wu P, Wang X, Zhou L, Tong Y, et al. Early Transmission Dynamics in Wuhan, China, of Novel Coronavirus–Infected Pneumonia. New England Journal of Medicine. 2020;382. doi:10.1056/NEJMoa2001316

3. The Novel Coronavirus Pneumonia Emergency Response Epidemiology Team. The Epidemiological Characteristics of an Outbreak of 2019 Novel Coronavirus Diseases (COVID-19) — China, 2020. CCDCW. 2020;2: 113–122. doi:10.46234/ccdcw2020.032

4. Chen LY, Ho KY, Phua KH. Normal bowel habits and prevalence of functional bowel disorders in Singaporean adults--findings from a community based study in Bishan. Community Medicine GI Study Group. Singapore Med J. 2000;41: 255–258.

5. Fang X, Lu S, Pan G. [An epidemiologic study of bowel habit in adult non-patient population in Beijing area]. Zhonghua Yi Xue Za Zhi. 2001;81: 1287–1290.

6. Rendtorff RC, Kashgarian M. Stool patterns of healthy adult males. Diseases of the Colon & Rectum. 1967;10: 222–228. doi:10.1007/BF02617184

7. Wood S. mgcv: Mixed GAM Computation Vehicle with Automatic Smoothness Estimation. 2022. Available: https://CRAN.R-project.org/package=mgcv

8. HKTDC. Wuhan (Hubei) City Information. In: HKTDC Research [Internet]. [cited 13 Mar 2023]. Available: https://research.hktdc.com/en/data-and-profiles/mcpc/provinces/hubei/wuhan

9. Macrotrends. Macrotrends - The Premier Research Platform for Long Term Investors. [cited 15 Mar 2023]. Available: https://www.macrotrends.net

10. Worldwide OA. Flight Database & Statistics | Aviation Analytics | OAG. [cited 13 Mar 2023]. Available: https://www.oag.com

11. Worldwide routes and flights from all airports - FlightsFrom.com. [cited 13 Mar 2023]. Available: https://www.flightsfrom.com/

12. Zhang Y, Cen M, Hu M, Du L, Hu W, Kim JJ, et al. Prevalence and Persistent Shedding of Fecal SARS-CoV-2 RNA in Patients With COVID-19 Infection: A Systematic Review and Meta-analysis. Clinical and Translational Gastroenterology. 2021;12: e00343. doi:10.14309/ctg.0000000000000343
