# Supplementary material for: Feasibility of wastewater-based detection of emergent pandemics through a global network of airports
Source: PLOS Glob Public Health. 2024 Mar 13;4(3):e0003010. doi: 10.1371/journal.pgph.0003010 (PMC10936834; doi:10.1371/journal.pgph.0003010)
Supplement: S2 Text — Further information on the derived detection probabilities and delay in modal detection times due to subsampling in the main analysis. (DOCX) [file pgph.0003010.s002.docx]

**S2_Text: Appendix containing additional details on results for the main analysis**

We consider the following four different time points [1]:

1. 30 December 2019, when pneumonia cases of unknown origin were reported to China National Health Commission;
2. 7 January 2020, when the novel coronavirus was isolated;
3. 13 January 2020, when the first SARS-CoV-2 case outside China (in Thailand) was reported;
4. 22 January 2020, the day before the epicentre, Wuhan, was locked down.

Table A. Probabilities (%) of an airport reporting positive wastewater samples from Wuhan by different time points—30 December 2019, 7 January 2020, 13 January 2020, and 22 January 2020, assuming wastewater was tested from all inbound flights. Destination airports are sorted by the detection probabilities.

| **Destination**  **airport** | **City** | **30 Dec 2019** | **7 Jan 2020** | **13 Jan 2020** | **22 Jan 2020** |
| --- | --- | --- | --- | --- | --- |
| **PEK** | Beijing | 0.31 | 2.20 | 9.3 | 59.6 |
| **PVG** | Shanghai | 0.15 | 1.12 | 4.8 | 36.7 |
| **NRT** | Tokyo | 0.07 | 0.51 | 2.2 | 18.8 |
| **CDG** | Paris | 0.04 | 0.23 | 1.3 | 15.4 |
| **SIN** | Singapore | 0.06 | 0.40 | 1.8 | 15.1 |
| **BKK** | Bangkok | 0.05 | 0.35 | 1.6 | 13.4 |
| **HKG** | Hong Kong | 0.04 | 0.27 | 1.2 | 10.4 |
| **ICN** | Seoul | 0.03 | 0.24 | 1.1 | 9.5 |
| **TPE** | Taipei | 0.03 | 0.21 | 0.9 | 8.0 |
| **JFK** | New York | 0.04 | 0.24 | 1.4 | 7.4 |
| **DXB** | Dubai | 0.04 | 0.24 | 1.4 | 7.4 |
| **FRA** | Frankfurt | 0.01 | 0.22 | 0.2 | 6.8 |
| **LHR** | London | 0.03 | 0.18 | 1.0 | 5.5 |
| **LAX** | Los Angeles | 0.02 | 0.13 | 0.7 | 4.1 |

Table B. Delays in modal detection times (in days) when the authority randomly samples only 100$p\%$ of all inbound planes at the airports, compared to the baseline scenario in which all inbound planes are sampled at the network of 20 destination airports. Comparisons were done for Wuhan as well as the four alternative epicentres (Madrid, Miami, Mombasa, and Mumbai), but all the scenarios were run for only 45 (Wuhan) or 50 (the four alternative epicentres) days, assuming these are pre-lockdown periods when infection and cross-border travel occur.

| $\mathbf{P}\{\mathrm{Sampled}\}$ **(%)** | **Wuhan**  **(China)** | **Madrid**  **(Spain)** | **Miami**  **(the US)** | **Mombasa**  **(Kenya)** | **Mumbai**  **(India)** |
| --- | --- | --- | --- | --- | --- |
| **50** | 0 | 3 | 3 | 3 | 3 |
| **20** | 3 | 7 | 7 | 6 | 7 |
| **10** | 3 | 9 | 10 | 8 | 10 |


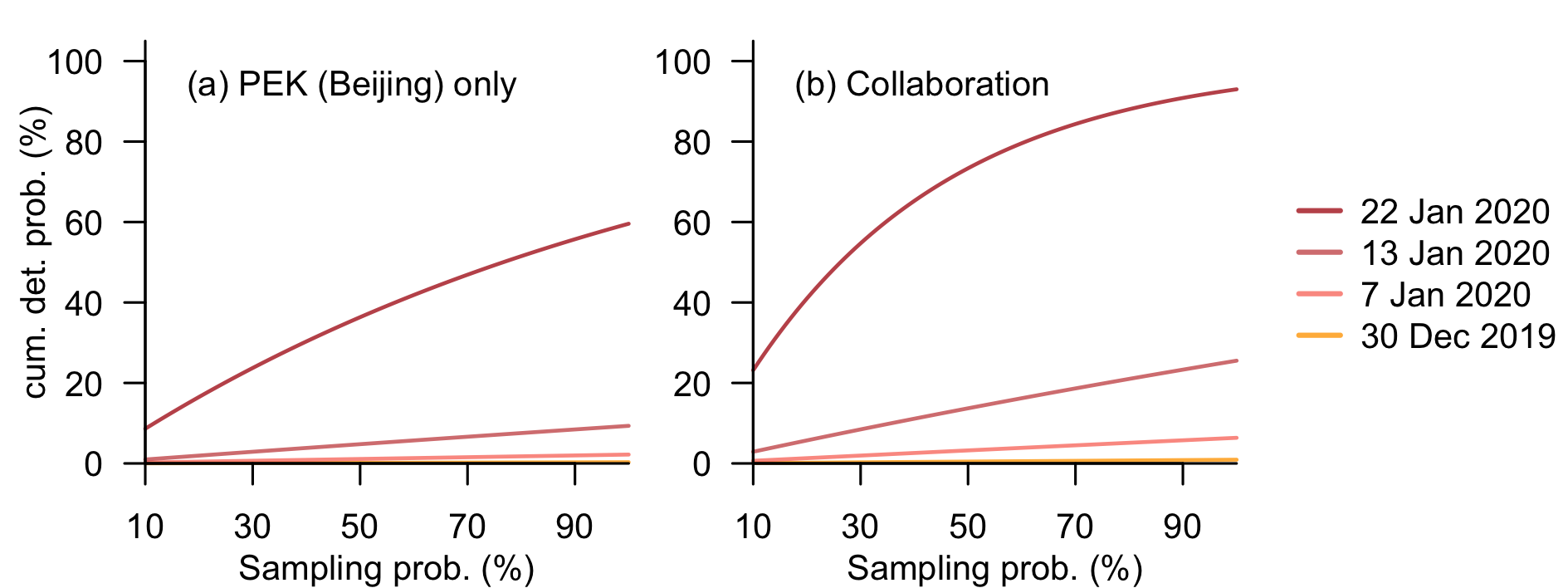


Fig A. Association between sampling probability and the corresponding cumulative detection probability at four time points for two scenarios: (a) wastewater was tested from different proportions of all inbound flights to Beijing Capital International Airport (PEK) which operated its own surveillance system; (b) wastewater was tested from different proportions of inbound flights to the collaborating network of 20 major airports in Asia, Europe and North America.


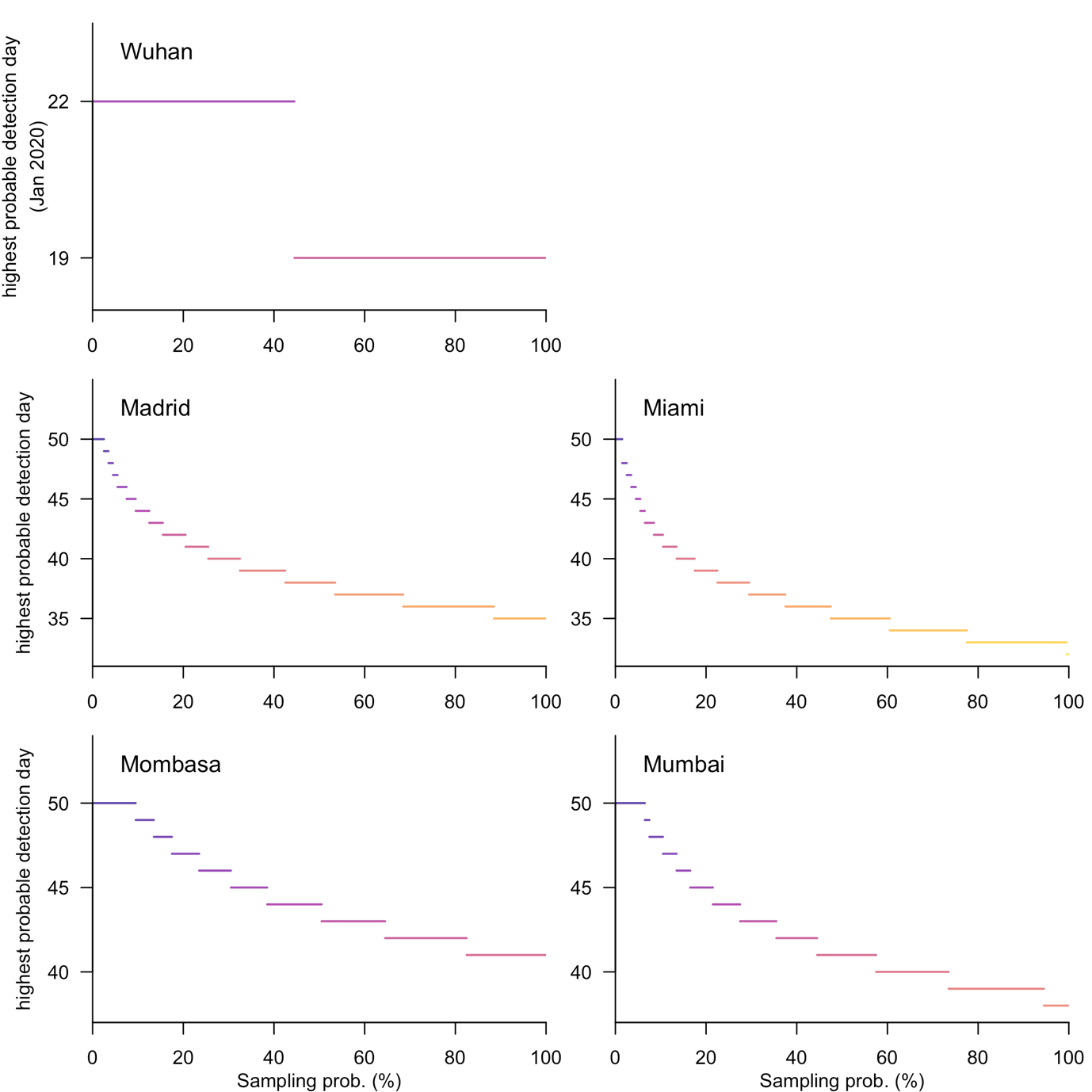


Fig B. Association between sampling probability and the corresponding highest probable detection days, assuming the 20 major airports collaborated on inbound aircraft wastewater surveillance. Different colours of the lines are for different days with the highest new detection probabilities, but are the same across different (hypothetical) epicentres.

**Reference**

1. Wang C, Horby PW, Hayden FG, Gao GF. A novel coronavirus outbreak of global health concern. Lancet. 2020;395: 470–473. doi:10.1016/S0140-6736(20)30185-9
