# Supplementary material for: Feasibility of wastewater-based detection of emergent pandemics through a global network of airports
Source: PLOS Glob Public Health. 2024 Mar 13;4(3):e0003010. doi: 10.1371/journal.pgph.0003010 (PMC10936834; doi:10.1371/journal.pgph.0003010)
Supplement: S3 Text — A list of sensitivity analyses performed for alternative sets of parameters or scenarios considered for probability calculation. (DOCX) [file pgph.0003010.s003.docx]

**S3_Text. Appendix containing sensitivity analyses**

**Impact of stochasticity in infection size on the aggregated detection probabilities**

In the main analysis, we assumed the actual infection size were known and fixed. However, stochasticity might exist, and we would like to know its impact on estimation results.

Let $q_{t}$ be the modelled probability of infection for an individual in community at time $t$ (i.e., the number of new infections, $I_{t}\sim Binom(N, q_{t})$, where $N$ was the total population. Following the central limit theorem, we presumed the probability of viral shedding at time $t$, $\sum_{s=1}^{20} I_{t-s}/N{=:p}_{t}^{v}\sim N(\sum_{s=1}^{20} q_{t-s}, \sum_{s=1}^{20} q_{t-s}(1-q_{t-s})/N)$. We simulated 100 000 different $p_{t}^{v}$s for each day $t$ and calculated time series of the cumulative probability of viral detection. We considered the baseline scenario of the COVID-19 outbreak in Wuhan and assumed wastewater from all the inbound flights were tested at the 20 major airports.


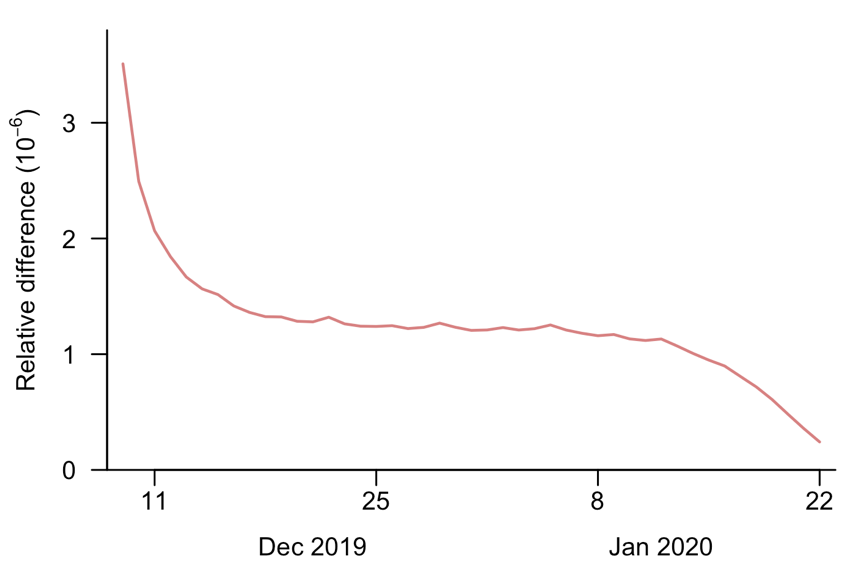


Fig A. Relative difference in the cumulative detection probabilities against time. Relative difference refers to the difference between the upper and lower bound of the 95% confidence interval divided by the point estimate (mean).

**Reduced toilet use on board**

If the average probability for one to use toilet on board is $\gamma$ ($\leq1$) times of that on land (the usual time), the probability defaecation for an individual during the time interval $[T_{1},T_{2}]$ would then be

$p_{[T_{1},T_{2}]}^{d}=\sum_{n=1}^{4} f_{def}\left( n \right)\cdot\min\{1, \int_{[T_{1},T_{2}]} \gamma f_{def}^{n}\left( s | n \right)ds\}$.

We considered three cases: $\gamma=0.5, 0.2$ and $0.1$ [1], assuming all the inbound planes were sampled at the network of the 20 major airports, and compared with the case when people were equally likely to defaecate on land and on board. Results were similar to those with reduced sampling probabilities (Fig B, Table A).

Table A. Delays in modal detection times (in days) when the average probability for one to use toilet (defaecate) on board is $100p\%$ of that on land, compared to the baseline scenario in which we assume air travel does not affect their toileting habits. Comparisons were done for Wuhan as well as the four alternative epicentres (Madrid, Miami, Mombasa, and Mumbai), but all the scenarios were run for only 45 (Wuhan) or 50 (the four alternative epicentres) days, assuming these are pre-lockdown periods when infection and cross-border travel occur.

| $\mathbf{P}\{\mathrm{Def}a\mathrm{ecation}\}$ **(%)** | **Wuhan**  **(China)** | **Madrid**  **(Spain)** | **Miami**  **(the US)** | **Mombasa**  **(Kenya)** | **Mumbai**  **(India)** |
| --- | --- | --- | --- | --- | --- |
| **50** | 0 | 3 | 3 | 3 | 3 |
| **20** | 3 | 6 | 7 | 6 | 7 |
| **10** | 3 | 9 | 10 | 9 | 10 |


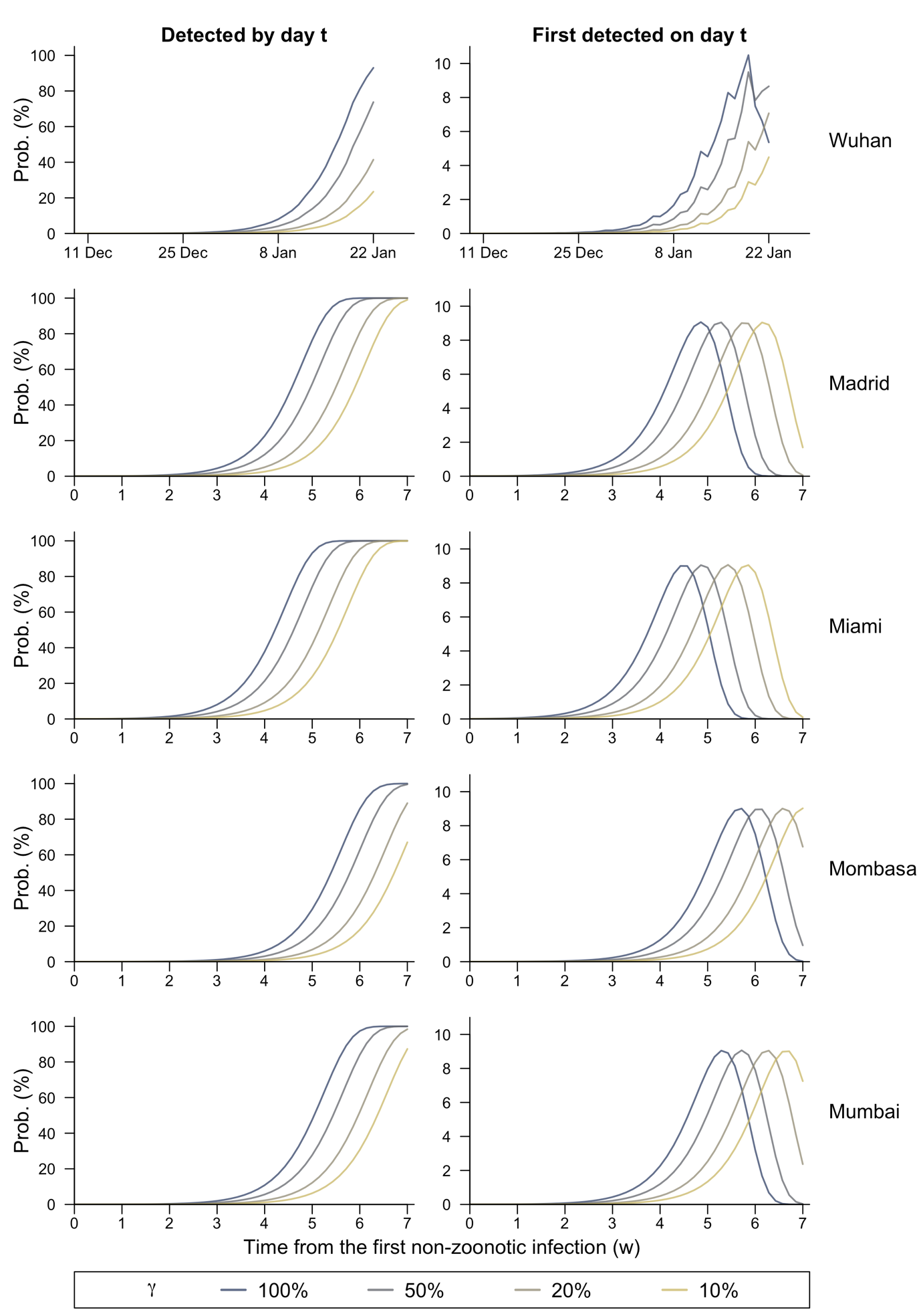


Fig B. Detection probability against time for different epicentres. Probability of the virus having been detected by day $t$ (column 1) or first detected on day $t$ (column 2) under four cases with diverse relative probabilities of defaecation on board (100%, 50%, 20%, 10%), assuming different epicentres—Wuhan, Madrid, Miami, Mombasa, and Mumbai—and routine aircraft wastewater surveillance for all the inbound flights at the 20 airports investigated. The scenario with Wuhan as the epicentre was run until 22 January 2020, the day before the city went into lockdown, while the other four had a pre-lockdown delay of 50 days.

**Ban from toilet use during the last 30 minutes of the flight**

Generally, aircraft lavatories are closed during taxi, climbing (takeoff) and descending (landing). While toilet visits that should have taken place shortly before (taxi) and after (climbing) the aircraft takes off are usually postponed until it is cruising, those that should have taken place when the aircraft is descending are often postponed until the passengers reach the airport terminals. Therefore, we consider a sensitivity analysis for the case that passengers are not allowed to use aircraft lavatories 30 minutes before arriving at destination airports. The results are slightly different from those for the case that passengers can use aircraft lavatories at any time during the flight (Fig C, Table B).


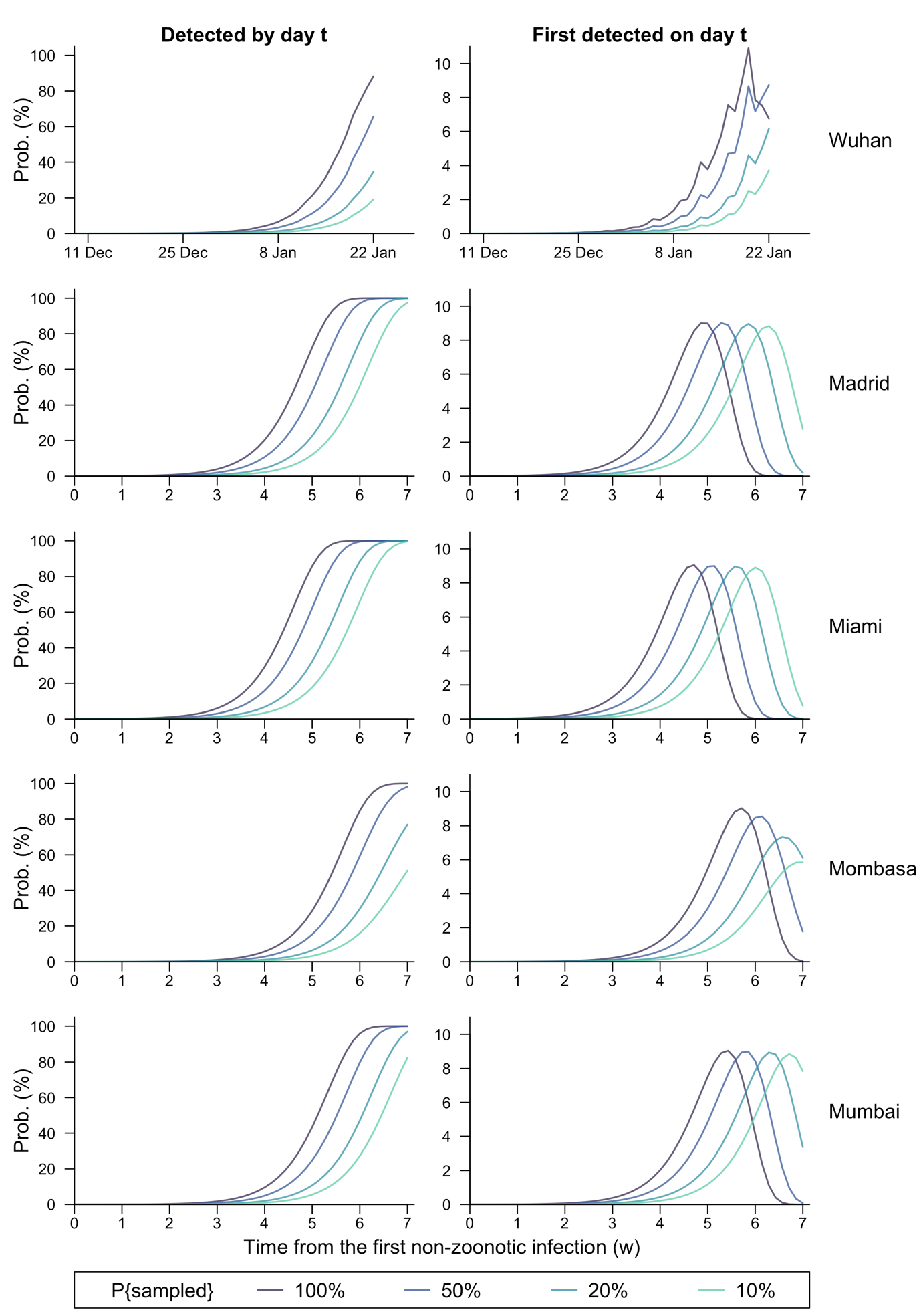


Fig C. Detection probability against time for different epicentres. Probability of the virus having been detected by day t (column 1) or first detected on day t (column 2) under four cases with diverse sampling probabilities (100%, 50%, 20%, 10%) for inbound flights, assuming different epicentres—Wuhan, Madrid, Miami, Mombasa, and Mumbai—and routine aircraft wastewater surveillance at all the 20 airports investigated. A ban from toilet use for the last 30 minutes of the flights was also taken into account. The scenario with Wuhan as the epicentre was run until 22 January 2020, the day before the city went into lockdown, while the other four had a pre-lockdown delay of 50 days.

Table B. Delays in modal detection times (in days) when passengers are not allowed to use toilets during the last 30 minutes of the flight, compared to the baseline scenario in which we assume passengers can access the toilets at any time during the flight. Comparisons were done for Wuhan as well as the four alternative epicentres (Madrid, Miami, Mombasa, and Mumbai), but all the scenarios were run for only 45 (Wuhan) or 50 (the four alternative epicentres) days, assuming these are pre-lockdown periods when infection and cross-border travel occur.

| **Wuhan**  **(China)** | **Madrid**  **(Spain)** | **Miami**  **(the US)** | **Mombasa**  **(Kenya)** | **Mumbai**  **(India)** |
| --- | --- | --- | --- | --- |
| 0 | 0 | 2 | 0 | 1 |

**Different departure time for the flights**

To test the potential impacts of departure time of the flights on the estimated detection probabilities, we alternatively calculated the probabilities by averaging over all the possible departure times of flights in a day (i.e., from 0:00 to 23:59). Nevertheless, we did not change the specific days for the flights (i.e., day-of-the-week arrangements) if they are not on a daily basis. The results are similar, but not identical to our previous estimates (Fig D).


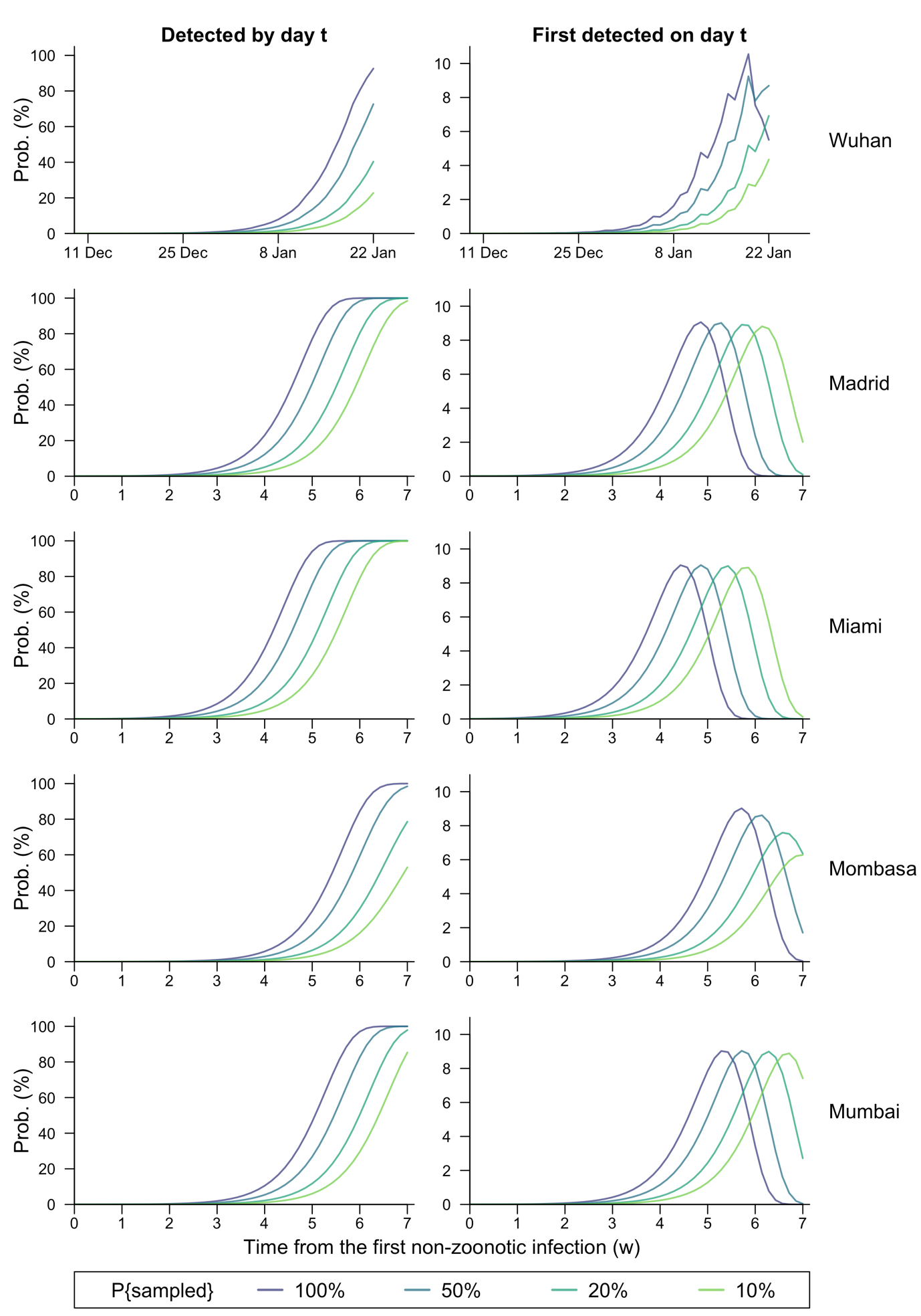


Fig D. Detection probability against time for different epicentres. Probability of the virus having been detected by day $t$ (column 1) or first detected on day $t$ (column 2) under four cases with diverse sampling probabilities (100%, 50%, 20%, 10%) for inbound flights, assuming different epicentres—Wuhan, Madrid, Miami, Mombasa, and Mumbai—and routine aircraft wastewater surveillance at all the 20 airports investigated. The detection probabilities were also averaged over all the possible departure times in a day. The scenario with Wuhan as the epicentre was run until 22 January 2020, the day before the city went into lockdown, while the other four had a pre-lockdown delay of 50 days.

**Changes in testing sensitivity**

In the main analysis, we assumed on average 50% of the stool samples from shedding individuals would be positive if tested. However, testing sensitivity may be virus-specific, or even change for different variants. Therefore, we conducted a sensitivity analysis assuming different rates of stools from shedding population being positive if tested—0.7, 0.5, 0.3, and 0.1. We only considered the baseline scenario in which wastewater from all inbound planes were tested at the network of 20 airports (Fig E, Table C).


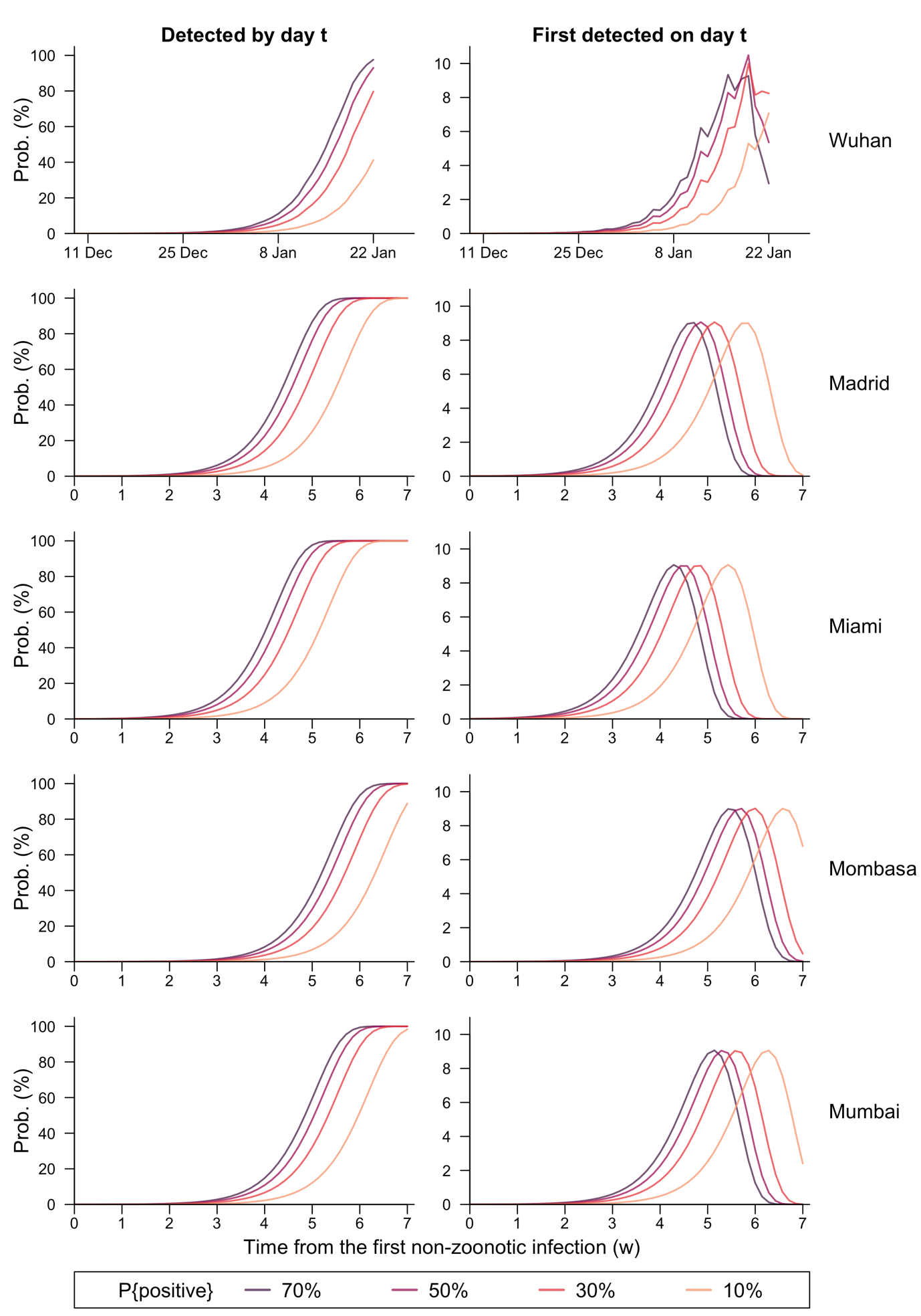


Fig E. Detection probability against time for different epicentres. Probability of the virus having been detected by day $t$ (column 1) or first detected on day $t$ (column 2) under four cases with diverse rates of stools from shedding population being positive (70%, 50%, 30%, 10%), assuming different epicentres—Wuhan, Madrid, Miami, Mombasa, and Mumbai—and routine aircraft wastewater surveillance for all inbound planes at all the 20 airports investigated. The scenario with Wuhan as the epicentre was run until 22 January 2020, the day before the city went into lockdown, while the other four had a pre-lockdown delay of 50 days.

Table C. Delays in modal detection times (in days) when the testing sensitivity (probability of stool sample from a shedding individual) being tested positive if tested) is some value between 0 and 1 other than 0.5, compared to the baseline scenario in which we assume infection can be detected through stool samples with a probability of 50%. Comparisons were done for Wuhan as well as the four alternative epicentres (Madrid, Miami, Mombasa, and Mumbai), but all the scenarios were run for only 45 (Wuhan) or 50 (the four alternative epicentres) days, assuming these are pre-lockdown periods when infection and cross-border travel occur.

| $\mathbf{P}\{\mathrm{Positivity}\}$ **(%)** | **Wuhan**  **(China)** | **Madrid**  **(Spain)** | **Miami**  **(the US)** | **Mombasa**  **(Kenya)** | **Mumbai**  **(India)** |
| --- | --- | --- | --- | --- | --- |
| **70** | -3 | -1 | -1 | -2 | -1 |
| **30** | 0 | 2 | 3 | 2 | 2 |
| **10** | 3 | 7 | 7 | 6 | 7 |

**Changes in the likelihood of travel for infections**

In the main analysis, we assumed people were equally likely to travel if infected. Nevertheless, the travelling pattern might be different for the infected population. Therefore, we conducted a sensitivity analysis assuming reduced probabilities of travel by 50%, 80%, and 90%. We only considered the baseline scenario in which wastewater from all inbound planes were tested at the network of 20 airports (see Table H in S1 Text). It should be noted that such changes in the likelihood of travel affect the detection probability in the same way as a change in the testing sensitivity.

Table D. Delays in modal detection times (in days) when infection reduces one’s likelihood to travel by $100\left( 1-p \right)\%$ (so that on average an infected individual is 100$p\%$ likely to travel compared to someone healthy), compared to the baseline scenario in which people are equally likely to travel if infected. Comparisons were done for Wuhan as well as the four alternative epicentres (Madrid, Miami, Mombasa, and Mumbai), but all the scenarios were run for only 45 (Wuhan) or 50 (the four alternative epicentres) days, assuming these are pre-lockdown periods when infection and cross-border travel occur.

| $\mathbf{P}\{\mathrm{Travel}\}$ **(%)** | **Wuhan**  **(China)** | **Madrid**  **(Spain)** | **Miami**  **(the US)** | **Mombasa**  **(Kenya)** | **Mumbai**  **(India)** |
| --- | --- | --- | --- | --- | --- |
| **50** | 0 | 3 | 3 | 3 | 3 |
| **20** | 3 | 7 | 7 | 6 | 7 |
| **10** | 3 | 9 | 10 | 9 | 10 |

**Sampling medium- and long-haul flights only**

Prior research has established that longer flights play an important role in successful viral detection in aircraft wastewater [2]. To assess the impact of medium- and long-haul flights (i.e., flights with a duration of at least three hours) in our models, we conducted a sensitivity analysis in which we exclusively sampled all such flights. This scenario was applied to all five (hypothetical) epicentres.

Since flights from Mombasa and Mumbai to the 20 major airports all exceeded three hours in duration, chances of detecting the virus were not affected by such an exclusion (Fig F, Table E). However, in the case of Wuhan, where most outbound flights to these 20 airports had durations shorter than three hours, excluding short-haul flights from testing would have significantly reduced the detection probability (Table F). This indicates substantial contribution of short-haul flights to successful viral detection, despite a higher probability of defaecation on board during longer flights.


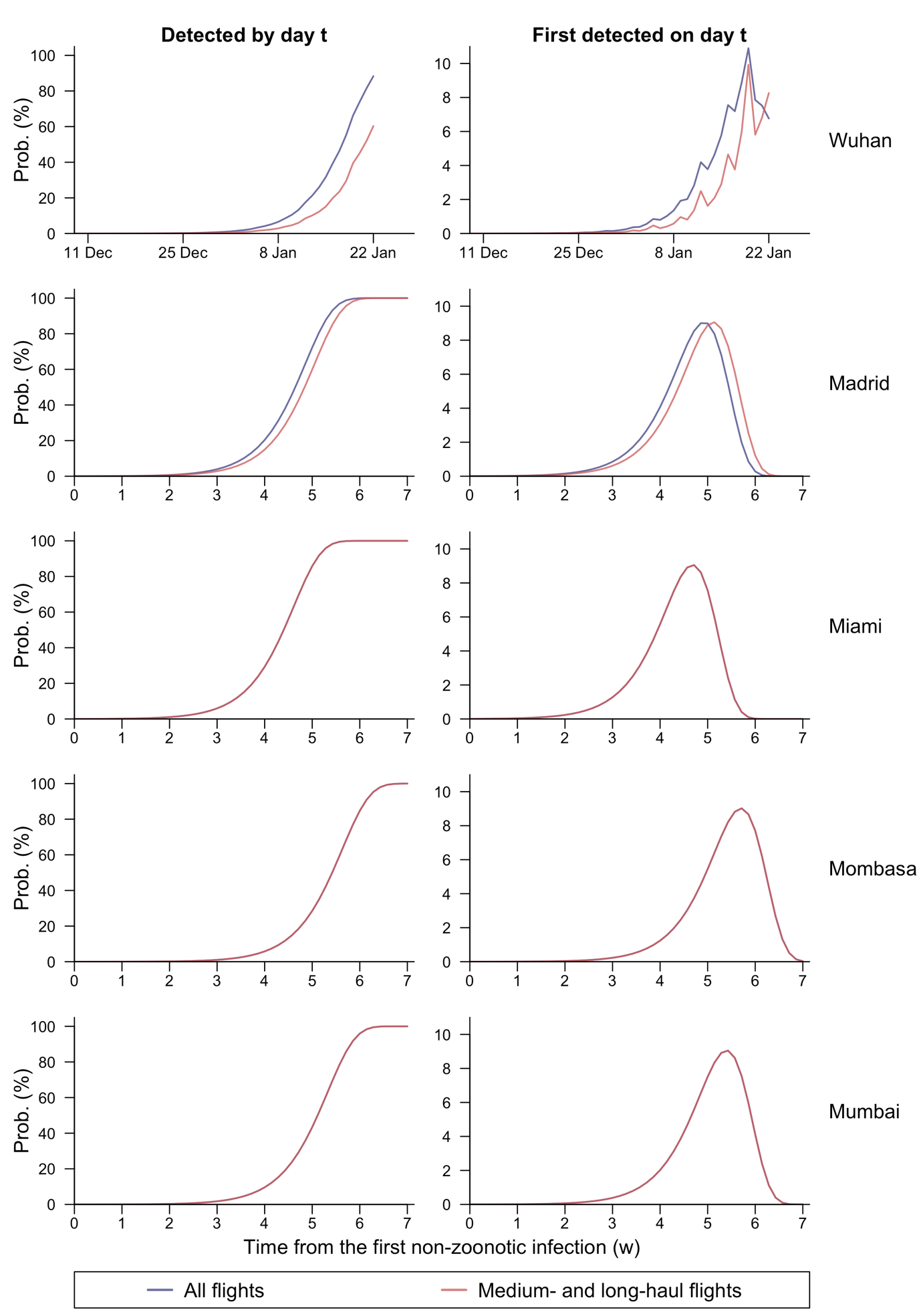


Fig F. Detection probability against time for different epicentres. Probability of the virus having been detected by day $t$ (column 1) or first detected on day $t$ (column 2) with (blue) or without (red) wastewater sampling from short-haul flights, assuming different epicentres—Wuhan, Madrid, Miami, Mombasa, and Mumbai—and routine aircraft wastewater surveillance for all inbound planes at all the 20 airports investigated. The scenario with Wuhan as the epicentre was run until 22 January 2020, the day before the city went into lockdown, while the other four had a pre-lockdown delay of 50 days.

Table E. Delays in modal detection times (in days) when wastewater from only medium- and long-haul flights were tested, compared to the baseline scenario in which we sampled all the inbound flights at all the 20 major airports. Comparisons were done for Wuhan as well as the four alternative epicentres (Madrid, Miami, Mombasa, and Mumbai), but all the scenarios were run for only 45 (Wuhan) or 50 (the four alternative epicentres) days, assuming these are pre-lockdown periods when infection and cross-border travel occur.

| **Wuhan**  **(China)** | **Madrid**  **(Spain)** | **Miami**  **(the US)** | **Mombasa**  **(Kenya)** | **Mumbai**  **(India)** |
| --- | --- | --- | --- | --- |
| 0 | 2 | 1 | 0 | 0 |

Table F. Percentage reduction in the probabilities of the 20 major airports reporting positive wastewater samples from Wuhan, Madrid, and Miami (epicentres with short-haul flights to the 20 major airports) by different time points—end of the third to the seventh week since the start of the outbreak—assuming wastewater was tested from all inbound medium- and long-haul flights.

| **Time from outbreak (w)** | **Wuhan***  **(China)** | **Madrid**  **(Spain)** | **Miami**  **(the US)** |
| --- | --- | --- | --- |
| **3** | 0.4 | 1.1 | 0.9 |
| **4** | 2.4 | 5.5 | 3.8 |
| **5** | 12 | 14 | 4.3 |
| **6** | 32 | 1.2 | 0 |
| **7** | N.A. | 0 | 0 |

*Time series of modelled infection sizes and detection probabilities for Wuhan were truncated by 22 January 2020 (day 45), since people were banned from leaving the city except for special reasons since the city was locked down on 23 January 2020.

**References**

1. Hertzberg VS, Weiss H, Elon L, Si W, Norris SL, FlyHealthy Research Team. Behaviors, movements, and transmission of droplet-mediated respiratory diseases during transcontinental airline flights. Proc Natl Acad Sci U S A. 2018;115: 3623–3627. doi:10.1073/pnas.1711611115

2. Shingleton JW, Lilley CJ, Wade MJ. Evaluating the theoretical performance of aircraft wastewater monitoring as a tool for SARS-CoV-2 surveillance. PLOS Glob Public Health. 2023;3: e0001975. doi:10.1371/journal.pgph.0001975
